# Lower diastolic tension may be indicative of higher proarrhythmic propensity in failing human cardiomyocytes

**DOI:** 10.1038/s41598-024-65249-0

**Published:** 2024-07-29

**Authors:** Xin Zhou, Paul Levesque, Khuram Chaudhary, Myrtle Davis, Blanca Rodriguez

**Affiliations:** 1 Department of Computer Science, University of Oxford, Wolfson Building, Parks Road, Oxford, OX1 3QD UK; 2 Discovery Toxicology, Bristol Myers Squibb, Lawrenceville, NJ USA

**Keywords:** Heart failure, Diastolic tension, Contractility, Arrhythmia, Modelling, Electromechanics, Ventricular tachycardia, Computational models

## Abstract

Chronic heart failure is one of the most common reasons for hospitalization. Current risk stratification is based on ejection fraction, whereas many arrhythmic events occur in patients with relatively preserved ejection fraction. We aim to investigate the mechanistic link between proarrhythmic abnormalities, reduced contractility and diastolic dysfunction in heart failure, using electromechanical modelling and simulations of human failing cardiomyocytes. We constructed, calibrated and validated populations of human electromechanical models of failing cardiomyocytes, that were able to reproduce the prolonged action potential, reduced contractility and diastolic dysfunction as observed in human data, as well as increased propensity to proarrhythmic incidents such as early afterdepolarization and beat-to-beat alternans. Our simulation data reveal that proarrhythmic incidents tend to occur in failing myocytes with lower diastolic tension, rather than with lower contractility, due to the relative preserved SERCA and sodium calcium exchanger current. These results support the inclusion of end-diastolic volume to be potentially beneficial in the risk stratifications of heart failure patients.

## Introduction

Heart failure (HF) is a complex disorder characterized by the inability of the heart to pump blood efficiently, which is one of the most common reasons for hospitalization. About half of the cardiac related deaths in chronic HF patients arise from progressive pump dysfunction, and the other half die suddenly and unexpectedly from ventricular tachyarrhythmias^1^. Although low left ventricular ejection fraction (LVEF) is clinically used in risk stratification for the need of defibrillator devices^2^, many patients who suffer from sudden cardiac death have preserved LVEF^3^. In addition to LVEF, larger end-diastolic volume biomarkers have been reported to be associated with cardiovascular events^4^, and increased mortality^5,6^ .This suggests the involvement of end-diastolic volume could improve current arrhythmic risk stratifications. However, the mechanistic link between contractility, relaxation and arrhythmic risk is unclear.

Many cardiovascular conditions promote the pathogenesis of chronic HF, including myocardial infarction, hypertension, congenital cardiomyopathies, myocarditis, cardiotoxicity, with additional risk factors such as aging, obesity, diabetes, kidney diseases and others. Maladaptive changes in the expression and function of several proteins with key roles in myocardial excitation–contraction coupling contribute to the loss of contractile function, diastolic dysfunction and lethal tachyarrhythmias in heart failure. Electrophysiological remodelling has been identified experimentally at the cellular level, including the alterations of sarcolemmal potassium, calcium, sodium currents, fluxes through pumps and exchangers (e.g. SERCA, ryanodine receptors), as well as regulatory proteins such as CaMKII^7^.

It is not completely clear how ionic current remodeling in HF contribute to the pathological phenotypes of the failing myocytes, especially the relationship between proarrhythmic abnormalities and electromechanics. Identifying the electromechanical consequences of HF remodelling is challenging due to the complexity of non-linear dynamic systems involved. Human-based computer modelling and simulations integrate and expand experimental data and knowledge from ionic currents to whole organ dynamics. Previous work on chronic HF modelling and simulations mainly focused on the electrophysiological effects of HF ionic current remodelling in failing myocyte or tissue^8–10^, while the electromechanical coupling and contractility, the heterogeneity and severity in HF remodeling, were not explored.

The goal of this study is to conduct advanced computer modelling and simulation studies to investigate the HF ionic current remodelling underlying the loss of contractility, the diastolic dysfunction and the proarrhythmic abnormalities in failing cardiomyocytes, considering different severity and variability using populations of human ventricular cell electromechanical models, calibrated and validated with human experimental data. We aim to investigate the relationship between the contractile function and the proarrhythmic abnormalities, as this would provide insights for the linkage between arrhythmic risk and LVEF which is currently used in clinical risk stratification.

## Methods

### Baseline healthy human ventricular cell model of electromechanics

The latest ToR-ORd model of human ventricular electrophysiology^11^ was coupled with the Land human contractility model^12^ as the baseline model, as in Margara et al.^13^. To enable the reproduction of enhanced calcium-activated potassium current (I_KCa_), and junctional sarcoplasmic reticulum calcium leak (J_leak_JSR_), new components of I_KCa_ and J_leak_JSR_ were introduced to the baseline model as illustrated in the Supplementary Material SM1.

With the coupling of the Land model, the calcium buffering was weaker than in the ToR-ORd model, leading to faster calcium transient kinetics and lower diastolic calcium level when SERCA was inhibited (Supplementary Material SM2, Supplementary Fig. S1). Based on the features of troponin C in the electromechanical coupling (Supplementary Material SM2, Supplementary Fig. S2), two modifications were made in the electromechanical model: (1) the effect of troponin C high affinity C-domain was included with K_D_ = 3 nM (Supplementary Material SM2, Supplementary Fig. S3); (2) K_D_ value for the binding of calcium and the N-domain of troponin C was decreased from 0.805 to 0.5 µM (Supplementary Material SM2, Supplementary Fig. S4). However, these changes also led to increased peak active tension and slower time to peak for active tension. The MatLab function *ga* was used to optimise the amplitude and kinetics of active tension (Ta) in both the healthy normal zone (NZ) and under SERCA inhibition (Supplementary Material SM2, Supplementary Fig. S5). This new ToR-ORd-Land model produced similar action potential as the ToR-ORd model and was able to produce early afterdepolarization (EAD), alternans, and predict drug responses in range with experimental data (Supplementary Material SM3, Supplementary Figs. S6–S9).

### Construction of the electromechanical models of human failing cardiomyocytes

Following myocardial infarction, ionic current remodelling occurs both in infarct border zone around the scar and in the remote myocardium. In our study, post-infarction border zone (BZ) and remote zone (RZ) were modelled separately for the investigation of electromechanical heterogeneity in the failing hearts. We considered two models of ionic current remodeling in the post-infarction border zone (BZ1-2) and one type of remote zone (RZ1) based on post-infarction minipig failing myocyte data as well as some failing human myocyte measurements^14–16^. Another type of RZ remodeling (RZ2) was established based on multiple experimental references of ischemic or dilated human cardiomyopathic failure^14,15,17–21^. The two types of BZs and RZs have different extents of SERCA inhibition based on the experimental reports that elevated wall stress adjacent to scars can result in down-regulation of SERCA^22^. HF ionic current remodeling (Table 1) leads to the reduction of the fast sodium current conductance (G_Na_), the L-type calcium current conductance (G_CaL_), the transient outward potassium current conductance (G_to_), the rapid/slow delayed rectifier potassium current conductances (G_Kr_, G_Ks_), the inward rectifier potassium current conductance (G_K1_), the sodium/potassium pump permeability (G_NaK_) and the SERCA activity (P_Jup_), as well as the increase in the late sodium current conductance (G_NaL_), the calcium-activated potassium and chloride current conductances (G_KCa_, G_ClCa_), the increase in CaMKII phosphorylation rate (aCaMK) with a slower calcium release process in the ryanodine receptors (Tau_relp), and the enhanced junctional sarcoplasmic reticulum calcium leak (J_leak_JSR_).

**Table 1:** BZ and RZ ionic remodeling of individual currents in HF remodelling.

Scaling factors	BZ1	BZ2	RZ1	RZ2
G_Na_	0.43^15^	0.43^15^	0.43^15^	0.43^15^
G_NaL_	1.275^16^	1.275^16^	1.413^16^	2^15,19^
G_to_				0.6^18,23^
G_CaL_	0.7^16^	0.7^16^		
G_Kr_	0.89^16^	0.89^16^	0.87^16^	0.6^24^
G_Ks_				0.4^18^
G_K1_	0.76^16^	0.76^16^		0.6^18,23^
G_NaK_				0.6^17^
P_Jup_	0.4^14,21^	0.3^14,21^	0.4^14,21^	0.3^14,21^
G_KCa_	2^16^	2^16^	2^16^	3.75^20^
G_ClCa_	1.25^16^	1.25^16^	1.25^16^	1.25^16^
aCaMK	1.5^25^	1.5^25^	1.5^25^	1.5^25^
Tau_relp	6^26^	6^26^	6^26^	6^26^
G_JSR_Leak_	4^27^	4^27^	4^27^	4^27^

### Construction, calibration and validation of the healthy and HF populations of human electromechanical models

An initial population of 2000 human ventricular endocardial cell models was constructed based on the new ToR-ORd-Land model by varying the G_Na_, G_NaL_, G_to_, G_CaL_, G_Kr_, G_Ks_, G_K1_, G_NCX_ (the sodium calcium exchanger conductance), G_NaK_, P_Jrel_ and P_Jup_ by up to ± 50% using Latin hypercube sampling. The endocardial cell models were paced at 1 Hz and calibrated by accepting those producing simulated action potential (AP), calcium transient (CaT) and active tension (Ta) biomarkers in range with human experimental measurements (Supplementary Material SM4, Supplementary Table S1). The endocardial models in the calibrated population were then used to produce epicardial and midmyocardial models by altering parameters as in the ToR-ORd model^11^, and models producing EAD, repolarization failure, or depolarization failure at 1 Hz, 2 Hz, 2.5 Hz and 3 Hz were discarded. The final accepted population of models contains 244 healthy models consistent with human experimental recordings, as shown in Supplementary Material SM4, Supplementary Fig. S10. Although only active tension amplitude was used in the calibration of the population of healthy models, the other active tension biomarkers were also consistent with experimental data (Supplementary Material SM4, Supplementary Fig. S11).

HF ionic current remodelling listed in Table 1 was applied to the 244 models in the calibrated healthy population, yielding the corresponding HF populations of electromechanical myocyte models. The relative biomarker changes from healthy to HF were computed for the baseline model and the population of electromechanical models, and validated against experimental ranges, as described in the Results section.

### Simulation platform, protocol and calculation of biomarkers

Cellular electromechanical simulations, Latin hypercube sampling, two-sided Wilcoxon rank sum tests (alpha = 0.05) and Pearson partial correlation analysis were performed using MATLAB codes. All the electromechanical myocyte models were paced at 1 Hz, 2 Hz, 2.5 Hz and 3 Hz for 300 beats to detect EAD and alternans generation. Action potential and calcium transient durations at 90% or 50% recovery were computed as APD_90_, APD_50_, CaTD_90_ and CaTD_50_, which reflect the length from excitation–contraction to recovery-relaxation for action potential and calcium transients. In addition, the peaks of action potential, calcium transient and active tension were computed as V_max_, CaT_max_ and Ta_max_, while their diastolic values were calculated as the resting membrane potential (RMP), CaT_min_ and Ta_min_. Calcium and active tension amplitudes were defined as the difference between their peak and diastolic values. Time to peak (TTP), and peak time to 50%, 90% and 95% of decay (RT50, RT90 and RT95) were also calculated for active tension as measurements of relaxation speed. A ΔAPD_90_ greater than 3 ms between the last two beats at steady state was defined as alternans.

## Results

### Validation of the HF electromechanical myocyte models

**Figure 1:**
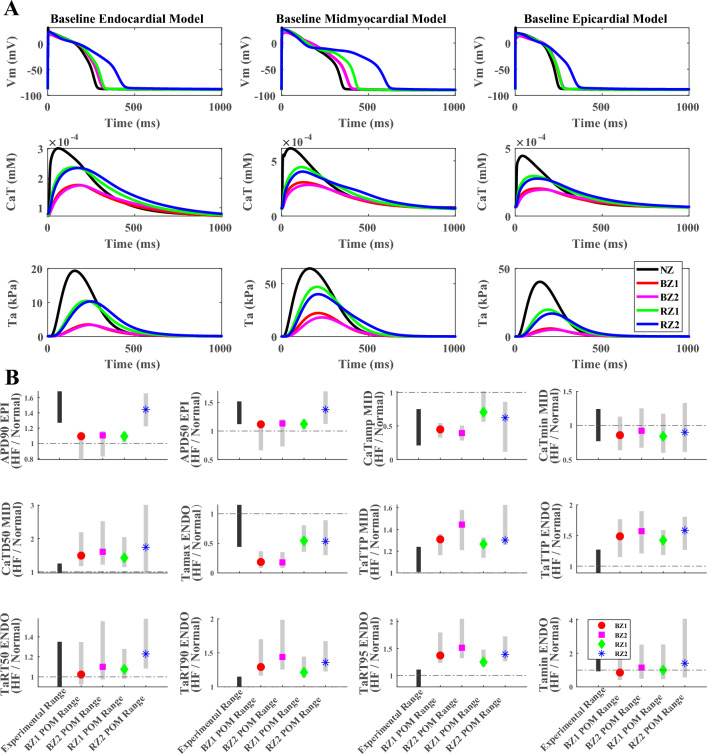
HF ionic current remodelling reproduced action potential, calcium transient and active tension biomarkers in range with experimental observations. (**A**) Effects of 4 types of HF ionic current remodelling on the membrane potential (Vm), calcium transient (CaT) and active tension (Ta) traces of endocardial, midmyocardial and epicardial baseline models. (**B**) Validation of the relative biomarker changes of action potential, calcium transient and active tension (HF/NZ) against experimental observations. The black bars cover the experimental ranges of biomarker variations, while the grey bars are distributions of biomarker changes from the population of models. The baseline model biomarkers are labelled as circles (BZ1), squares (BZ2), diamonds (RZ1), and asterisks (RZ2).

As illustrated in Fig. 1A, the HF ionic remodeling induced longer action potential duration (APD), weaker calcium transient and reduced peak active tension than healthy myocytes. The activation and decay were slower in the HF calcium transient and active tension. Diastolic calcium and active tension tended to be elevated, corresponding to diastolic dysfunction and slower relaxation in HF. While all four subtypes of HF remodelling led to longer action potential than the healthy model, the RZ2 caused the most severe action potential prolongation, as a result of the strongest potassium current inhibitions in the four types of HF remodelling. The two RZs had larger peaks of calcium transient and active tension than the two BZs due to the lack of I_CaL_ inhibition in the RZs. BZ1 tended to have stronger and earlier peaks of calcium transient and active tension than the corresponding BZ2, suggesting the crucial role of SERCA difference in the regulation of calcium dynamics.

Simulated action potential, calcium transient and active tension biomarkers from the HF models were validated against experimental data ranges as shown in Fig. 1B. Prolongation of action potentials, calcium transients, and active tension kinetics (as biomarker changes in APD_90_, APD_50_, CaTD_50_, TaTTP, TaRTs) observed in failing cardiomyocytes were reproduced in the simulations, and the reduction in amplitudes of calcium transient and active tension as well as the enhanced diastolic tension reported in HF were also generated in the simulations. Due to the scarcity of the human heart tissue samples, the calculation of experimental ranges may be limited since only a few studies were included and the number of cells involved in the data was small, especially for APD_90_, APD_50_, TaTTP, TaRTs, and Ta_min_, with details listed in Supplementary Material SM4, Supplementary Table S2^18,21,28–32^. Nevertheless, quantitative agreements were observed in the comparison of APD_90_, APD_50_, CaT_amp_, CaT_min_, Ta_max_, TaRT_50_ and Ta_min_ for all or some subpopulations of HF models, with most experimental data considered in this validation step not used for model construction and calibration.

### Enhanced I_NaL_ and suppressed I_Kr_ are the main drivers of action potential prolongation in HF myocytes

**Figure 2:**
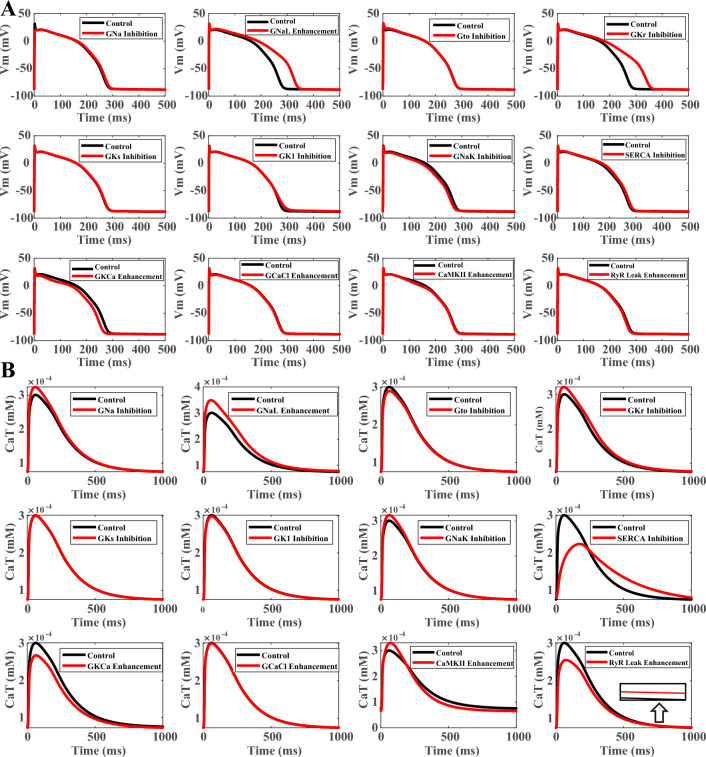
Contribution of individual HF ionic current remodelling to the morphology and duration of membrane potential (**A**), the loss of calcium transient peak and the elevation of diastolic calcium (**B**).

After the evaluation of the HF biomarkers, the contribution of each ionic current remodelling on the alteration of biomarkers was analyzed in Fig. 2. The inhibited I_Na_ contributed most to the loss of action potential peak. The augmentation of I_NaL_ and the weaker I_Kr_ contributed most to the prolongation of action potential in failing cardiomyocytes, while the weaker I_NaK_, and the I_KCa_ enhancement mildly counteracted the action potential prolongation in HF (Fig. 2A).

### Remodeling of both sarcoplasmic reticulum calcium dynamics and sarcolemmal currents contribute to the loss of contractility and the slower relaxation in failing myocytes

Similar analysis was conducted to the calcium transient and active tension of HF models. As illustrated in Fig. 2B, HF remodeling of different ionic currents and fluxes contributed to the alteration of calcium transient in HF. SERCA inhibition and the augmented junctional sarcoplasmic reticulum calcium leak through ryanodine receptors (RyR) were the biggest sources for the loss of calcium amplitude, and similar trend was observed in active tension (Supplementary Material SM5, Supplementary Fig. S12A). While the enhanced I_KCa_ and weaker I_to_ also contributed to weaker contractility, stronger I_NaL_, weaker I_Na_, I_Kr_ and I_NaK_, as well as increased CaMKII activity all prevented the loss of contractility (Fig. 2B, Supplementary Fig. S12). SERCA inhibition was the biggest contributor to the slower decay and diastolic elevation of calcium transient and active tension. While I_NaL_ enhancement and I_Kr_ inhibition mildly contributed to the slower relaxation, stronger I_KCa_ and CaMKII both promoted faster relaxation (Fig. 2B, Supplementary Fig. S12A). The modulation of calcium dynamics by I_KCa_, I_NaL_, I_Kr_ and I_Na_ was achieved through their regulation of the action potential, whereby longer action potential prolonged the duration of the L-type calcium current and enhanced the calcium transient (Supplementary Material SM5, Supplementary Fig. S12B).

### Contribution of current variability to HF biomarkers

Partial correlation analysis was conducted to investigate the sensitivity of HF biomarkers to the ionic current conductances in the population of models. As shown in Supplementary Material SM5, Supplementary Fig. S13, stronger G_NaL_, G_NCX_ and weaker G_Kr_ were correlated to longer APD_90_ and APD_50_ in HF, while G_Na_, G_to_ and G_CaL_ may influence peak action potential (V_max_). Strong G_NaL_, weak G_Kr_, G_NCX_ and SERCA(P_Jup_) contributed to long CaTDs. Increased G_NaL_, G_CaL_, weaker G_NCX_, G_Kr_ promoted larger calcium transient and active tension amplitude, whereas suppressed G_NCX_ and SERCA led to diastolic calcium elevation (Supplementary Fig. S13). Weaker SERCA and stronger G_NCX_ contributed to longer activation time for active tension, while weaker SERCA was also related to longer active tension recovery time (Supplementary Fig. S13).

### HF ionic current remodelling promoted EAD generation through enhanced I_NaL_ and suppressed I_Kr_ in models with lower diastolic tension

**Figure 3:**
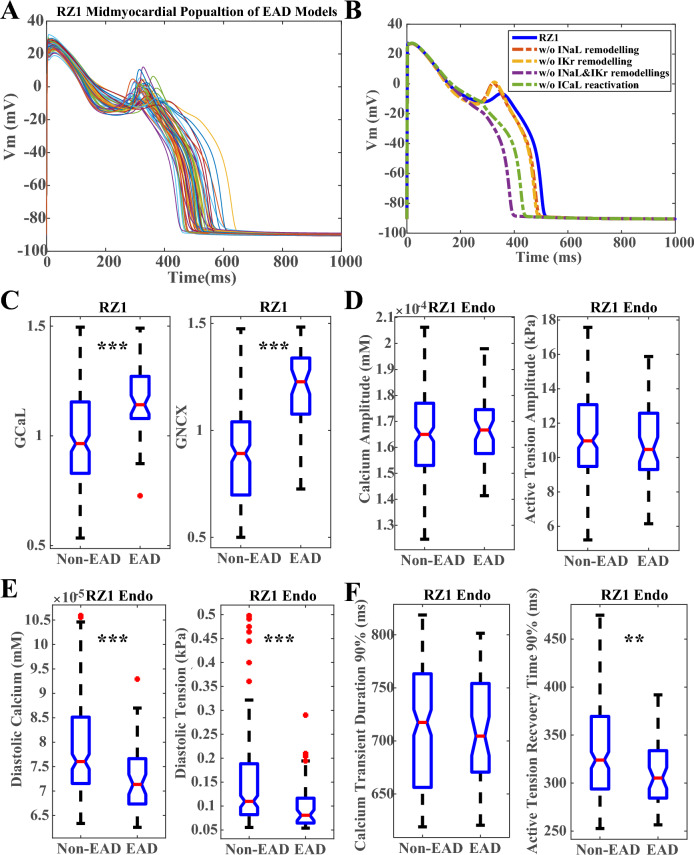
HF models with EAD generation in the midmyocardial layer tend to have similar contractility but lower diastolic calcium and tension. (**A**) Population of RZ1 midmyocardial models with EADs at 1 Hz pacing. (**B**) Removing I_NaL_ enhancement and I_Kr_ suppression eliminates EAD, whereas terminating I_CaL_ reactivation achieves the same. (**C**) EAD models tend to have stronger G_CaL_ and G_NCX_. Comparison of the amplitudes (**D**), diastolic levels (**E**) and durations (**F**) of the calcium and active tension in the corresponding endocardial models with and without EAD generation in the RZ1 midmyocardial layer. (***p < 0.001, **p < 0.01).

As HF patients often suffer from higher risk of ventricular arrhythmias, repolarization abnormalities, such as EADs, were assessed in the population of HF models. In the midmyocardial population of HF model, only one or two EADs were observed in the BZ populations at 1 Hz pacing. In the RZs, EADs were observed more frequently as illustrated in Fig. 3A. To demonstrate the effects of the individual HF ionic remodeling on the inducibility of EADs, a representative model was chosen from the RZ1 population with EAD (Fig. 3B). Removing the I_NaL_ remodeling did not eliminate the EAD (the orange dashed trace), and similarly when I_Kr_ inhibition was removed, EAD was still maintained (the yellow dashed trace). However, when both I_NaL_ enhancement and I_Kr_ suppression were absent, the EAD was eliminated (the purple dashed trace). Although I_NaL_ and I_Kr_ remodeling promoted EAD generation in HF, the initiation of EAD would not proceed without the re-activation of I_CaL_ (the green dashed trace). Similar mechanisms were also observed in RZ2 population as shown in the Supplementary Material SM6, Supplementary Fig. S14, where removing either I_NaL_ or I_Kr_ remodeling was enough to eliminate EAD.

In addition to I_NaL_ and I_Kr_ remodeling, variability in the population of models also contributes to the generation of EADs and other repolarization abnormalities (RA) such as repolarization failure. EAD and RA models tended to have stronger G_CaL_, G_NCX_ and weaker repolarization currents (Supplementary Material SM6, Supplementary Figs. S15–S16). Due to the stronger G_CaL_ and G_NCX_ (Fig. 3C), the corresponding endocardial and epicardial models of the RZ1 midmyocardial EAD models had similar amplitudes of calcium transient and active tension as the HF models without EADs (Fig. 3D). On the other hand, the stronger G_NCX_ in the EAD models also led to lower diastolic calcium and tension levels, as well as faster decay of the diastolic tension. (Fig. 3E,F). Similar findings were observed in the epicardial and RZ2 populations (Supplementary Material SM6, Supplementary Figs. S17-S19). HF models with EAD generation did not have more compromised contractility comparing to the non-EAD models, but they tended to have lower diastolic calcium and active tension, as well as faster recovery in tension due to the relative stronger sodium-calcium exchanger current. Therefore, a lower diastolic tension and larger diastolic volume in HF may contribute to higher arrhythmic risk.

### SERCA remodelling and CaMKII activation promote alternans generation in HF models with lower diastolic tension

**Figure 4:**
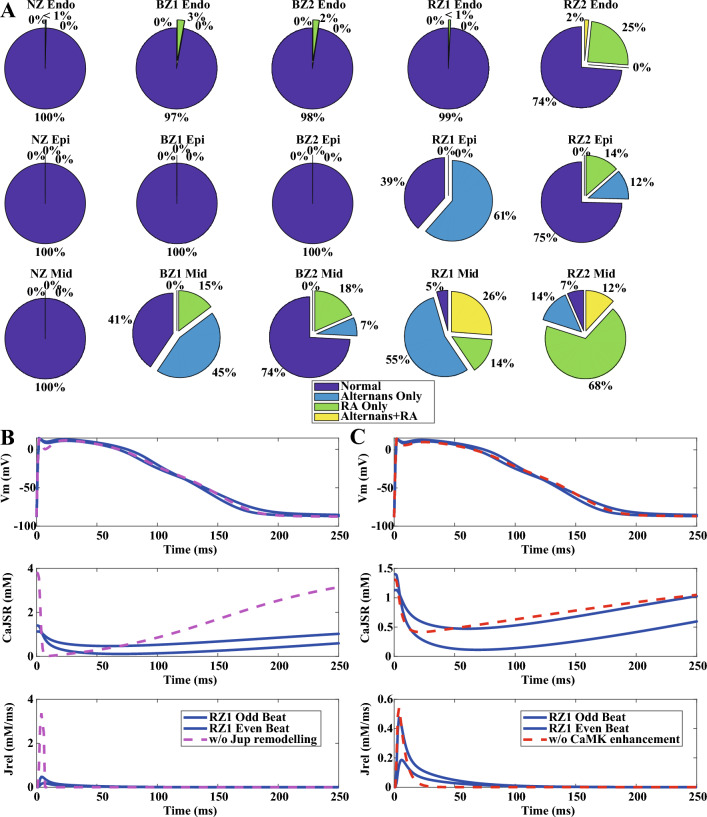
HF remodelling induced RA and alternans at fast pacing rates. (**A**) HF remodelling promoted alternans and RA in different population of HF models. (**B**,**C**) SERCA inhibition and CaMKII augmentation promoted alternans generation in HF models.

In addition to 1 Hz pacing, fast pacing of 2 Hz, 2.5 Hz and 3 Hz were applied to HF population of models to assess the generation of proarrhythmic abnormalities. Only one endocardial model in the healthy NZ calibrated population produced alternans. On the other hand, the HF populations developed repolarization abnormalities (RA) such as repolarization failure due to action potential prolongation, as well as beat-to-beat alternans at different pacing frequencies (Fig. 4A). The RZ populations of models tended to generate more alternans and repolarization abnormalities than the BZ populations, suggesting the role of preserved I_CaL_ in the initiation of RA and alternans.

To illustrate the contribution of HF ionic current remodelling, the biggest alternans at 2.5 Hz pacing from the RZ1 epicardial population was chosen as an example in Fig. 4. At CL = 400 ms, simulations exhibited alternations of long and short action potentials in the odd and even beats (blue solid traces), and it was clear that alternans was associated with the insufficient calcium re-uptake and slow calcium recovery in junctional sarcoplasmic reticulum (JSR). When the inhibition of SERCA pump (J_up_) was switched off (the magenta dashed traces), alternans disappeared along with a significant increase of the calcium level in the junctional sarcoplasmic reticulum (CaJSR, Fig. 4B). In addition to the insufficient calcium re-uptake, enhanced CaMKII and slower calcium release further contributed to the alternans. When CaMKII activation and calcium release (Jrel) kinetics were switched back to normal (the red dashed traces), the duration of calcium release was shorter, giving more time for junctional sarcoplasmic reticulum calcium to recover before the next beat, leading to the elimination of alternans (Fig. 4C).

**Figure 5:**
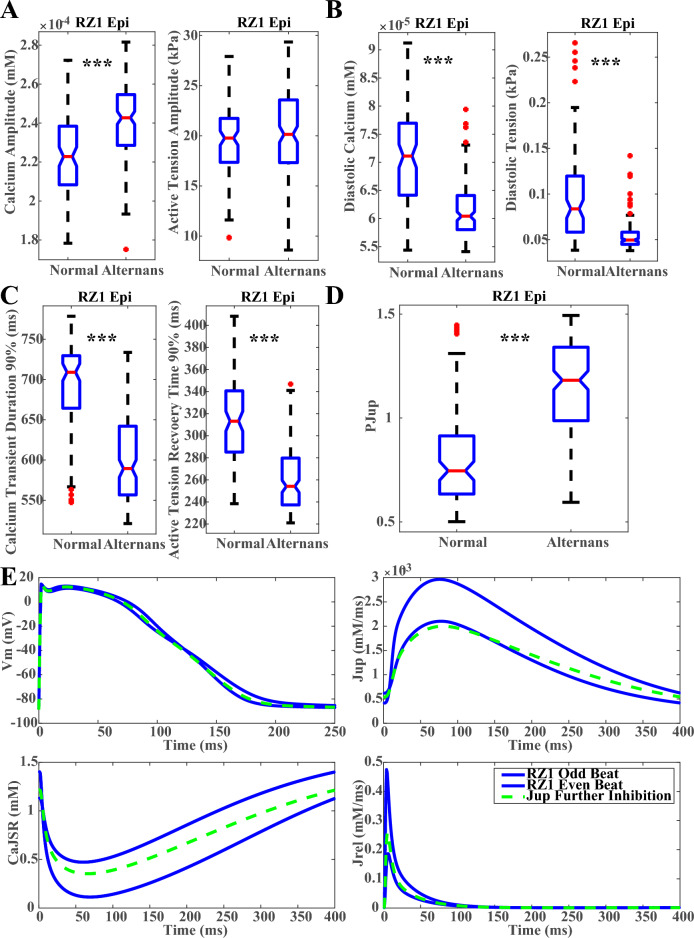
Alternans models tended to have more preserved SERCA function and lower diastolic tension. Comparison of the amplitudes (**A**), diastolic levels (**B**) and durations (**C**) of the calcium and active tension at 1 Hz with and without alternans generation in the RZ1 epicardial HF models. (**D**) Comparison of the P_Jup_ (SERCA) between the alternans and non-alternans models in the RZ1 epicardial models. (**E**) Further SERCA inhibition of 20% in the representative RZ1 epicardial alternans model eliminated the generation of alternans. (***p < 0.001).

Further analysis was conducted to assess the contractility of the alternans models at 1 Hz. For the BZ1 midmyocardial population and the RZ1 epicardial population, which generated most alternans at fast pacing with the size of alternans and normal populations comparable, the calcium amplitudes tended to be higher in the alternans models than in the non-alternans HF models at 1 Hz pacing (Fig. 5A, Supplementary Material SM6, Supplementary Fig. S20A), yielding similar or larger amplitude in the active tension (Fig. 5A, Supplementary Material SM6, Supplementary Fig. S20A). Alternans models consistently displayed lower diastolic calcium and tension levels, as well as shorter calcium and active tension durations than the non-alternans HF models (Fig. 5B,C, Supplementary Material SM6, Supplementary Fig. S20B,C). These phenomena were caused by the more preserved SERCA function in the alternans models (Fig. 5D, Supplementary Material SM6, Supplementary Fig. S20D). As illustrated in the Fig. 5E, when SERCA was further inhibited by 20% in the same alternans example of Fig. 4, the weaker calcium re-uptake led to lower initial calcium level in the sarcoplasmic reticulum (CaJSR) at the start of a beat, leading to a smaller calcium release and milder decrease of the junctional sarcoplasmic reticulum calcium level that was easier to refill before the start of the next beat (the green dashed traces). These results demonstrate that alternans models in the HF population could have more preserved SERCA function than the non-alternans HF models, corresponding to lower diastolic calcium and tension, which also suggests that the relatively faster diastolic relaxation and larger diastolic volume in HF may be associated with higher arrhythmic risk.

## Discussions

In this study, we systematically constructed, calibrated and validated populations of human electromechanical models of failing cardiomyocytes, and investigated the relationship between the contractility and the proarrhythmic abnormalities in the failing human electromechanical models. Our results show that proarrhythmic triggers tend to occur in failing myocyte models with lower diastolic tension and faster relaxation. Our simulation results provide digital evidence supporting the consideration of end-diastolic volume biomarkers in the improvement of current arrhythmic risk stratifications.

Both systolic and diastolic dysfunctions are typical conditions of HF, which sometimes coincide in a same patient. A clinical study which enrolled more than 200 patients revealed that severe diastolic dysfunction was associated with increased arrhythmic risk regardless of the degree of LVEF reduction^33^. In our HF cellular models, the augmentation of I_NaL_, and the inhibition of I_Kr_ and SERCA contribute to both diastolic dysfunction and cellular pro-arrhythmic EADs or alternans. On the other hand, we also observe that the cellular models with EADs or alternans were not the ones with the most severe elevation of diastolic tension, which suggests that other factors such as the presence of scars and fibrotic tissue favoring reentrant circuits may be the main contributors to arrhythmic risk in grade III diastolic dysfunction^33^.

**Figure 6:**
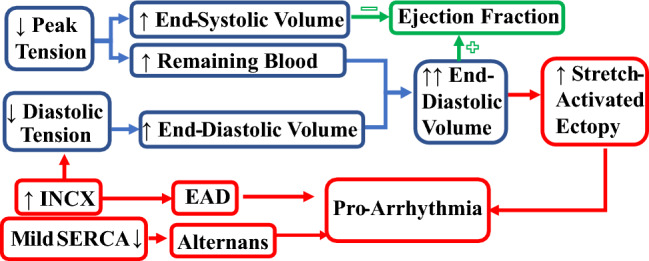
Effects of reduced peak and diastolic tension on ejection fraction, end-diastolic volume and arrhythmic risk.

A significant number of patients who suffer sudden cardiac death have relatively preserved LVEF^3^. As the increase of the end-diastolic volume and the decrease of the end-systolic volume both contribute to larger LVEF, the lower diastolic tension in the EAD and alternans models could be a factor contributing to higher end-diastolic volume in HF. The effect on LVEF however may be counteracted by the increase of end-systolic volume in clinical practice. In addition to the lower end-diastolic tension, the remaining blood volume in the ventricles caused by the compromised systolic contraction may contribute to further enlargement of the end-diastolic volume and trigger stretch-activated ectopic activity^34^ (Fig. 6). Larger end-diastolic volume was reported as an independent risk factor for cardiovascular events^4^, and increased mortality^5,6^. Our simulation results provide support for the consideration of end-diastolic volume biomarkers in the improvement of current arrhythmic risk stratifications.

In addition to the ionic current remodelling observed in ischemic or dilated cardiomyopathies, other factors also play roles in the pathogenesis of failing myocytes. For example, females tend to have lower expression of potassium channels and higher expression of calmodulin and calcium ATPase than males^35^, which may contribute to sex-specific differences in pro-arrhythmia as well as contraction and relaxation in HF. The elevation of diastolic tension seems to coincide with the rise of reactive oxygen species (ROS) and inflammation level^36,37^. Proinflammatory cytokines are known to promote ROS production, which leads to reduced nitric oxide bioavailability and protein kinase G activity which results in hypo-phosphorylation of titin and elevated resting tension^38^. On the other hand, HF induces inflammation via wall stress, and inflammatory cytokines can be induced in stretched myocytes/cardiac fibroblasts and in overloaded myocardium^39^. Future studies could be conducted to explore the effects of different therapeutic targets to break the vicious cycle.

In this study, we investigated the contributions of different ionic current remodelling and current variability on action potential prolongation, loss of contractility and diastolic dysfunction. Table 2 summarizes the roles of individual currents on action potential duration, peak calcium and active tension, diastolic calcium and active tension, EAD, and alternans. The results showed: (1) I_Kr_ inhibition and I_NaL_ enhancement played crucial roles in the prolongation of action potential and generation of EAD in HF; (2) SERCA(P_Jup_) inhibition and J_leak_JSR_ contributed most to the loss of calcium and active tension amplitudes in HF; (3) SERCA suppression was the biggest cause for the diastolic calcium and tension elevation; (4) SERCA remodelling and CaMKII activation induced alternans generation in failing myocytes.

**Table 2:** Summary of individual ionic current remodelling on the electromechanical properties of HF myocytes: red arrows indicate the ionic current remodelling caused maladaptive changes whereas the blue arrows mean the remodeling relieved adverse HF symptoms. ‘–’ means unaltered or insignificant effects. Purple arrows indicate currents which favored the abnormality generation if they were augmented.

Currents	HF remodelling	Action potential duration	Peak calcium and tension	Diastolic calcium and tension	EAD	Alternans
I_Na_		–		–	–	–
I_NaL_						–
I_to_		–		–	–	–
I_CaL_	or –	–		–		
I_Kr_						–
I_Ks_		–	–	–	–	–
I_K1_		–	–	–	–	–
I_NCX_	or –					–
I_NaK_				–		–
J_leak_JSR_		–			–	–
J_up_		–			–	
I_KCa_					–	–
I_ClCa_		–	–	–	–	–
CaMKII		–			–	

Based on the simulation results, I_Kr_ enhancement or I_NaL_ inhibition should be considered for the prevention of excessive action potential prolongation and EAD generation. Restoration of SERCA expression and function will improve both the contractility and relaxation of failing myocytes. CaMKII augmentation tended to promote higher calcium amplitude through its regulation on I_CaL_, however it also had the effect of inducing larger J_leak_JSR_ to lower JSR calcium content which then led to reduced calcium release^40^. The higher inducibility of alternans by stronger CaMKII activity also prevented it from being an ideal treatment target for HF.

In addition to the above remodelling, another two key currents also played important roles in the regulation of HF phenotypes with some existing controversies: I_CaL_ and I_NCX_. One controversy related to the remodelling of I_CaL_ in HF. Most studies reported no change in its current density in animal and human cardiomyocytes^18,28,41,42^. However, some experiments in smaller mammal species showed a reduction in I_CaL_ density^43^. Since I_CaL_ reduction was reported only in the BZ rather than the RZ of post-infarction heart failure minipigs^16^, we only applied I_CaL_ inhibition in the HF BZ models. As shown in Fig. 4A, the BZ models with I_CaL_ suppression tended to generate less alternans than the RZ models, which suggested that I_CaL_ reduction can be relevant for the inhibition of beat-to-beat alternans, consistent with previous research^44^. Due to the potential roles of I_CaL_ on the promotion of alternans and EADs, the augmentation of I_CaL_ is unlikely to be a safe strategy to recover contractility in HF without proarrhythmic risk, as shown by previous studies on calcium channel agonists^45^.

The remodelling of sodium-calcium exchanger in HF is also debatable. Some studies reported the increased expression of NCX in HF^42^, others argued the altered NCX activity could be the consequence of intracellular calcium and sodium variations^46,47^. Human and minipig studies reported no change in the rate of calcium removal from NCX, and no differences in I_NCX_ density^16,28^. As reviewed by Bers et al*.*^7^, an increase of NCX could compensate for a decrease of SERCA function, contributing to the maintenance of relative normal rates of calcium decline and relaxation. In this study, when we introduced SERCA inhibition without NCX enhancement, elevation of diastolic calcium and slower calcium decay was observed in the HF models, consistent with the group of HF patients who had reduced SERCA function but little alteration in NCX expression^48^. Although higher activity of NCX can contribute to normal diastolic function in HF, it can jeopardize systolic function and also increase the risk of action potential prolongation and EAD.

The restoration of SERCA expression and function is regarded as an effective strategy for the recovery of normal systolic and diastolic function. Efforts have been made to increase SERCA activity in treatment of HF, including with gene therapy in HF patients. Clinical trials of adeno-associated vector type 1 (AAV1)—mediated transgenic therapy of SERCA2a have improved cardiac function and prevented expansion of left ventricular volume^49^. However, larger clinical trial of AAV1-SERCA2a (CUPID2) did not show significant gains in the secondary endpoints, probably due to low gene delivery rate of SERCA2a^50^. Other regulatory strategies of SERCA function involve the direct post-translational modifications such as SUMOylation^51^, acetylation^52^, and phosphorylation^53^, as well as indirect regulations through phospholamban^54^. Our simulation results show that mild SERCA restoration in HF myocytes could maintain the risk of alternans generation. Wet-lab experiments and whole ventricular simulations could be conducted in future work to verify the phenomenon at the cellular and the organ level.

The regulation of diastolic calcium is less understood compared to changes in systolic calcium in HF. As reviewed by Eisner et al*.*^55^, the diastolic and systolic calcium regulations are closely linked. In addition to SERCA which takes up ~ 70% of the calcium, nearly 30% is extruded via I_NCX_ in the relaxation process^7^. In addition to SERCA, we show additional ionic current remodelling contributed to diastolic calcium elevation, such as the increased RyR leakage (J_leak_JSR_) and I_NaL_, and the inhibition of I_Kr_. Further studies can explore the interplay between increased intracellular sodium ions in HF and NCX activity, as well as the role of cytoplasmic calcium buffering in the regulation of systolic and diastolic functions.

To conclude, we systematically constructed, calibrated and validated population of human electromechanical models of failing cardiomyocytes. The simulation results demonstrated that proarrhythmic abnormalities can occur in failing myocytes with lower diastolic tension, suggesting the role of end-diastolic volume in risk stratifications.

### Supplementary Information

Supplementary Information.

## Data Availability

The datasets used and/or analysed during the current study are available from the corresponding author upon reasonable request.
