# Publisher Correction: Lower diastolic tension may be indicative of higher proarrhythmic propensity in failing human cardiomyocytes

**DOI:** 10.1038/s41598-024-70961-y

**Published:** 2024-08-27

**Authors:** Xin Zhou, Paul Levesque, Khuram Chaudhary, Myrtle Davis, Blanca Rodriguez

**Affiliations:** 1 Department of Computer Science, University of Oxford, Wolfson Building, Parks Road, Oxford, OX1 3QD UK; 2 Discovery Toxicology, Bristol Myers Squibb, Lawrenceville, NJ USA

Correction to: *Scientific Reports* 10.1038/s41598-024-65249-0, published online 29 July 2024

The original version of this Article contained errors in Figure 4, panels B and C, where the text in the legend box did not display correctly. The original Figure 4 and accompanying legend appear below.

**Fig. 4:**
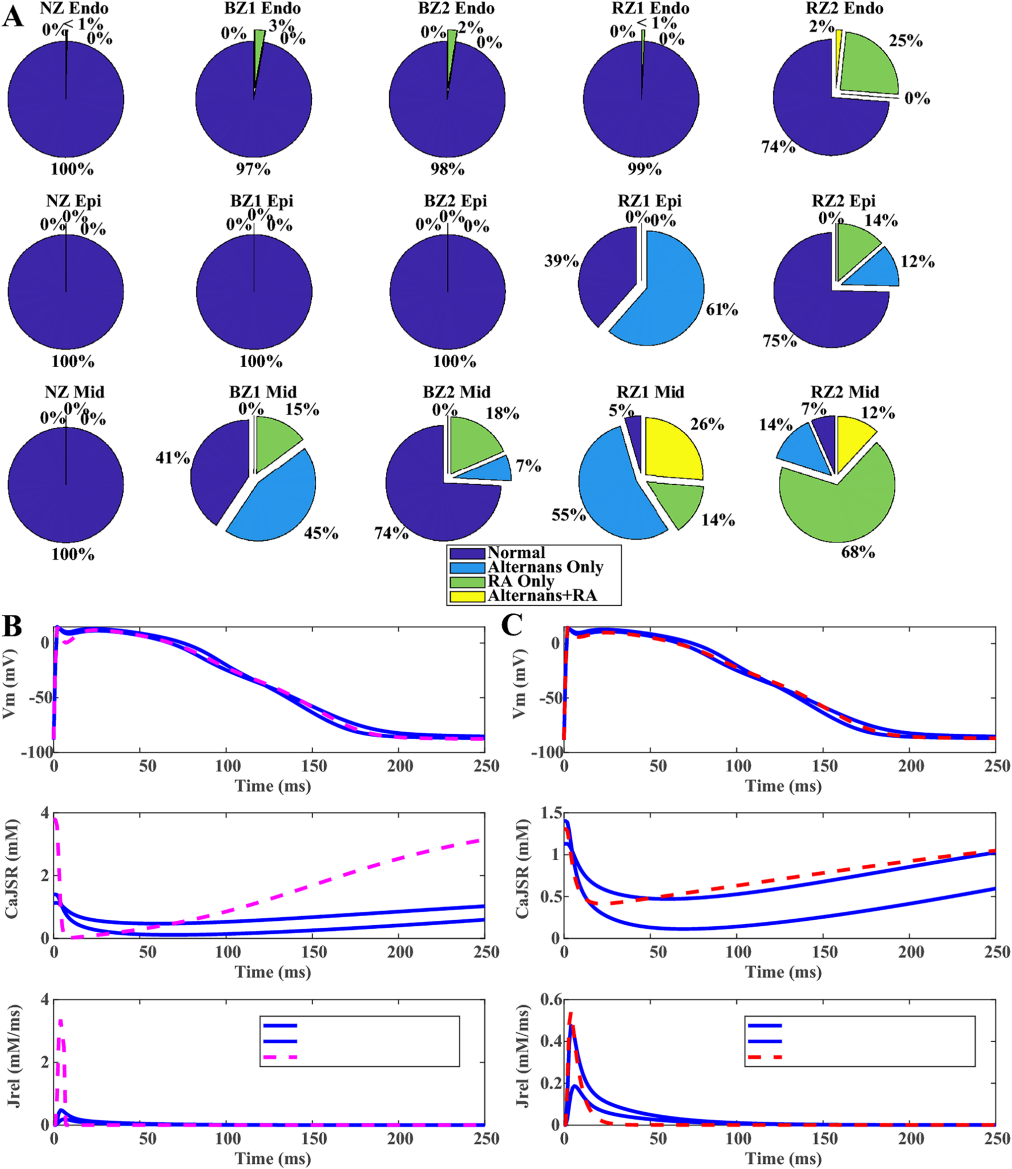
HF remodelling induced RA and alternans at fast pacing rates. (**A**) HF remodelling promoted alternans and RA in different population of HF models. (**B**,**C**) SERCA inhibition and CaMKII augmentation promoted alternans generation in HF models.

The original Article has been corrected.

