# Supplementary material for: Lower diastolic tension may be indicative of higher proarrhythmic propensity in failing human cardiomyocytes
Source: Sci Rep. 2024 Jul 29;14:17351. doi: 10.1038/s41598-024-65249-0 (PMC11286957; doi:10.1038/s41598-024-65249-0)
Supplement: Supplementary file 1 — Supplementary Information. [file 41598_2024_65249_MOESM1_ESM.docx]

**SUPPLEMENTARY MATERIALS**

**Table of Contents**

[**SM1: Implementation of the calcium activated potassium current (I_KCa_) and calcium leak from junctional sarcoplasmic reticulum (J_leak_JSR_) in the ToR-ORd model.** 2](#_Toc168063657)

[**SM2: Improvement of the electromechanical coupling between the ToR-ORd model and the Land model** 3](#_Toc168063658)

[**SM3: Validation of the new ToR-ORd-Land model with updates of I_KCa_, J_leak_JSR_ and the new Land model parameter fittings** 7](#_Toc168063659)

[**SM4: Construction, calibration and validation of the population of normal zone (NZ) and heart failure (HF) electromechanical models** 9](#_Toc168063660)

[**SM5: Contributions of individual ionic currents to HF phenotypes** 12](#_Toc168063661)

[**SM6: Contributions of ionic currents to the repolarization abnormality (RA) and alternans generation in HF models** 14](#_Toc168063662)

[**References** 19](#_Toc168063663)

**SM1: Implementation of the calcium activated potassium current (I_KCa_) and calcium leak from junctional sarcoplasmic reticulum (J_leak_JSR_) in the ToR-ORd model.**

As the calcium activated potassium current (I_KCa_) were reported to be enhanced in heart failure, a new formulation of the I_KCa_ was added into the ToR-ORd model based on the data by ^1^ to obtain an updated model. Due to the coupling of I_KCa_ channels and the L-type calcium channels^2^, the ratio of I_KCa_ channels in the subspace was set to be the same as the L-type calcium channels in the model. The conductance of I_KCa_ (gkca) was chosen to get a similar current density ratio between I_KCa_ and I_Kr_ as observed in minipig myocytes ^3^. The conductance of the background potassium current was scaled to 90% to adapt to the implementation of I_KCa_. The formulation of this new I_KCa_ current is the following:

gkca= 0.003;

ikcan=3.5;

kdikca=6.05e-04;

FractionI_KCa_ss=0.8;

FractionI_KCa_i=1-FractionI_KCa_ss;

I_KCa__ss=$\mathrm{gkca}\times\mathrm{Fraction}I_{KCa}ss\times\frac{{Ca}_{ss}^{ikcan}}{{Ca}_{ss}^{ikcan}+{kdikca}^{ikcan}}\times\left( Vm-EK \right)$ (1)

I_KCa__i=$\mathrm{gkca}\times\mathrm{Fraction}I_{KCa}i\times\frac{{Ca}_{i}^{ikcan}}{{Ca}_{i}^{ikcan}+{kdikca}^{ikcan}}\times\left( Vm-EK \right)$ (2)

I_KCa_=I_KCa__ss+I_KCa__i;

In order to produce the increased calcium leak from the junctional sarcoplasmic reticulum in heart failure, the following formulation was introduced in the ToR-ORd from ^4^ :

G_JSR_Leak = 1.7500e-04;

Km_JSR_Leak = 20;

Jleak_JSR = G_JSR_Leak $\times$ ${exp}^{\frac{\mathrm{Ca}\mathrm{jsr}}{Km\_JSR\_Leak}}$ $\times$ (Ca_jsr_ - Ca_ss_). (3)

**SM2: Improvement of the electromechanical coupling between the ToR-ORd model and the Land model**

Before the coupling to the Land model, the calcium buffering in the ToR-ORd model was:

$$\beta_{Cai}=\frac{1}{1+ \frac{\left[ \bar{CMDN} \right]K_{CMDN}}{\left( \left[ {Ca}^{2+} \right]_{i}+K_{CMDN} \right)^{2}}+ \frac{\left[ \bar{TRPN} \right]K_{TRPN}}{\left( \left[ {Ca}^{2+} \right]_{i}+K_{TRPN} \right)^{2}}}$$

(4)

With the coupling to the Land model, the troponin buffering was moved to the Land model, and the calcium buffering became:

$${\beta^{*}}_{Cai}=\frac{1}{1+ \frac{\left[ \bar{CMDN} \right]K_{CMDN}}{\left( \left[ {Ca}^{2+} \right]_{i}+K_{CMDN} \right)^{2}}}.$$

(5)

Therefore, the electromechanical coupling led to weaker calcium buffering and faster calcium transient kinetics. The amplified calcium decay rate was manifested as lower diastolic calcium level with the presence of SERCA inhibition as in shown in Figure S1.


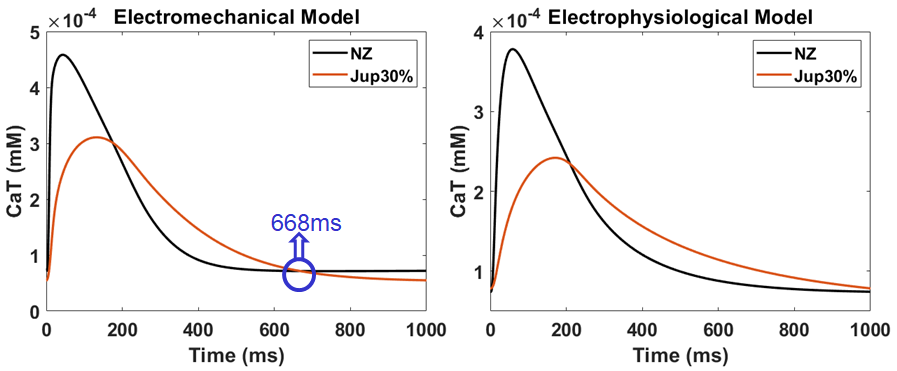


Figure S1: Effects of inhibiting SERCA activity (Jup 30%) in the electromechanical model (left) and the electrophysiological model (right) at 1Hz pacing. The contractility parameters of the electromechanical model were the same as in ^5^.

To fix this problem, two modifications were made in the electromechanical model. Firstly, the effect of troponin C high affinity C-domain was investigated. Troponin C is the key link between the calcium dynamics and the contractile apparatus in the excitation- contraction coupling process. Troponin C has three calcium binding domains, shown in Figure S2. The regulatory N-domain has a single low affinity calcium binding site, but it is the key calcium sensor of cardiac contraction. The K_D_ of N-domain varies between 0.3 - 2 µM ^6^. The structural C-domain contains two high affinity calcium binding sites, with the binding affinity varying between 3 - 40 nM ^6^.


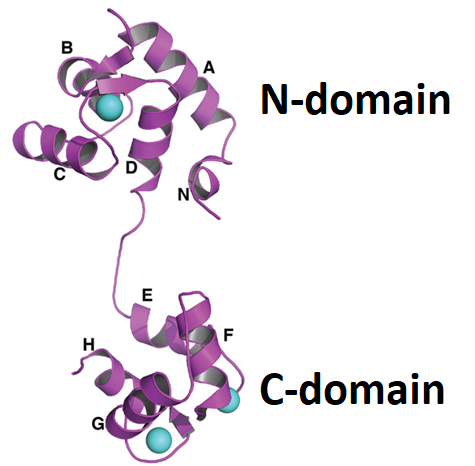


Figure S2: Structure of cardiac ToponinC·3Ca^2+^ (NMR, pdb 1AJ4). Ca^2+^ ions are shown as spheres ^6^.

Although the troponin C high affinity C-domain was regarded as always bound with calcium, when it was introduced into the electromechanical model with K_D_=3nM as in eq. (6), the calcium buffering capacity was increased, and SERCA inhibition led to diastolic CaT elevation before 907 ms of the pacing cycle (Figure S3).

$$\beta_{CaiNew}=\frac{1}{1+ \frac{\left[ \bar{CMDN} \right]K_{CMDN}}{\left( \left[ {Ca}^{2+} \right]_{i}+K_{CMDN} \right)^{2}}+ \frac{\left[ \bar{TRPNH} \right]K_{TRPNH}}{\left( \left[ {Ca}^{2+} \right]_{i}+K_{TRPNH} \right)^{2}}},$$

(6)

where K_TRPNH_=3e-6mM; $\bar{TRPNH}$=$\left[ \bar{TRPN} \right]\times$2.


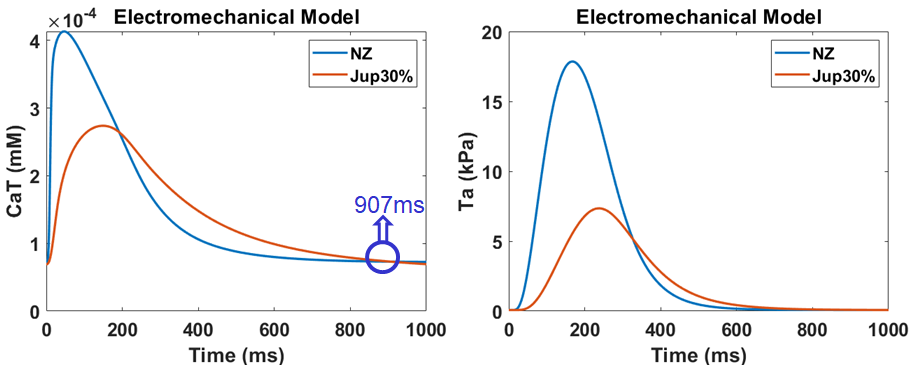


Figure S3: Introducing the effects of Troponin C high affinity binding sites in the C-domain increased calcium buffering capacity, and SERCA inhibition generated diastolic calcium elevation before 907 ms at 1Hz pacing (left). The increased diastolic active tension (Ta) was observed before 993 ms at 1Hz pacing.

Secondly, as variable K_D_ values were reported for the binding of calcium and the N-domain of troponin C, the effect of increasing calcium affinity in the N-domain was explored. As shown in Figure S4, increasing Troponin C calcium affinity in its N-domain also promoted diastolic calcium elevation under SERCA inhibition, and the elevation was observed before 891 ms of the 1Hz pacing (Figure S4, left). The augmentation of troponin-calcium sensitivity also had the effect of increasing active tension (Ta amplitude) and slowing its kinetics (Figure S4, right). Active tension peak was increased from 22 kPa to 38 kPa, and the time to peak for Ta (TaTTP) was slowed from 167 ms to 208 ms for the NZ model.


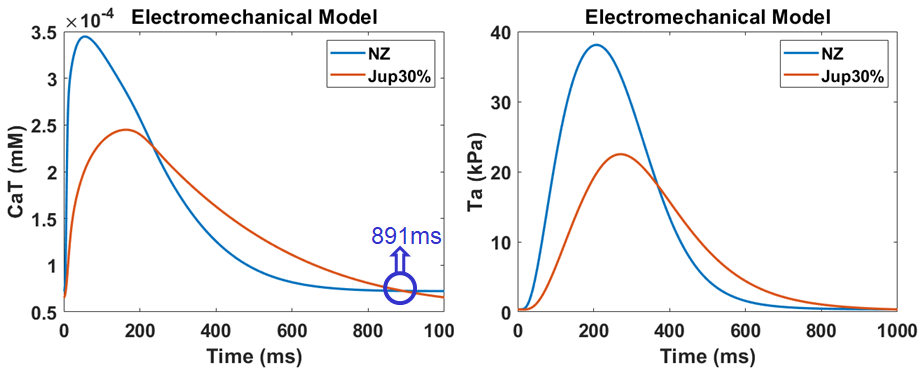


Figure S4: Increasing calcium affinity at the N-domain of troponin C by reducing half activation concentration from 0.805 µM to 0.5 µM facilitated the elevation of diastolic calcium before 891 ms (left) and produced higher Ta with slower kinetics (right). The increased diastolic Ta was observed before 986 ms at 1Hz pacing.

The combination of the above two changes in troponin-calcium buffering led to more significant diastolic calcium elevation under SERCA inhibition. To calibrate the new model, the following biomarkers were extracted from normal zone Ta: time to peak (TTP), peak time to 50% decay (RT50), peak time to 95% decay (RT95), maximum and minimum values. Ta from SERCA inhibition (Jup30%) was extracted as Ta2. Those values were fed into the following cost function:

$$d_{t}=d\left( TTP,\left[ 155,172 \right] \right)+d\left( RT50,\left[ 120,125 \right] \right)+d\left( RT95,\left[ 315,350 \right] \right)$$

$+10d\left( max(Ta),\left[ 22,25 \right] \right)+10d\left( max(Ta2),\left[ 8.4,13.3 \right] \right)$ (7)

To minimise the cost function, 4 contractility parameters which has the biggest effects on Ta from the Land model were varied. The MatLab function *ga* (with default parameters) was used, which finds the minimum of the function using a genetic algorithm. The updated parameters are the following:

perm50=0.4811;

ktm_unblock= 0.0311;

nperm=2.3252;

k_ws=0.0061;

The new electromechanical model is able to produce diastolic CaT and Ta elevation at 1Hz under SERCA inhibition (Figure S5), with the NZ Ta biomarkers in range with experimental observations ^7–9^.


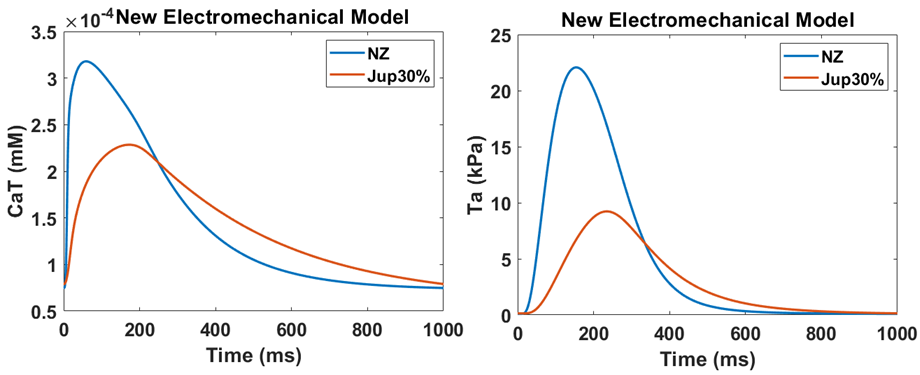


Figure S5: New electromechanical model with increased troponin C- calcium buffering generated diastolic CaT and Ta elevation under SERCA inhibition at 1Hz pacing. The elevation of diastolic CaT and Ta were observed throughout the pacing cycle.

**SM3: Validation of the new ToR-ORd-Land model with updates of I_KCa_, J_leak_JSR_ and the new Land model parameter fittings**

The new model produced similar action potential (AP) morphology as the ToR-ORd model, and can produce EADs and alternans (Figure S6-S8).


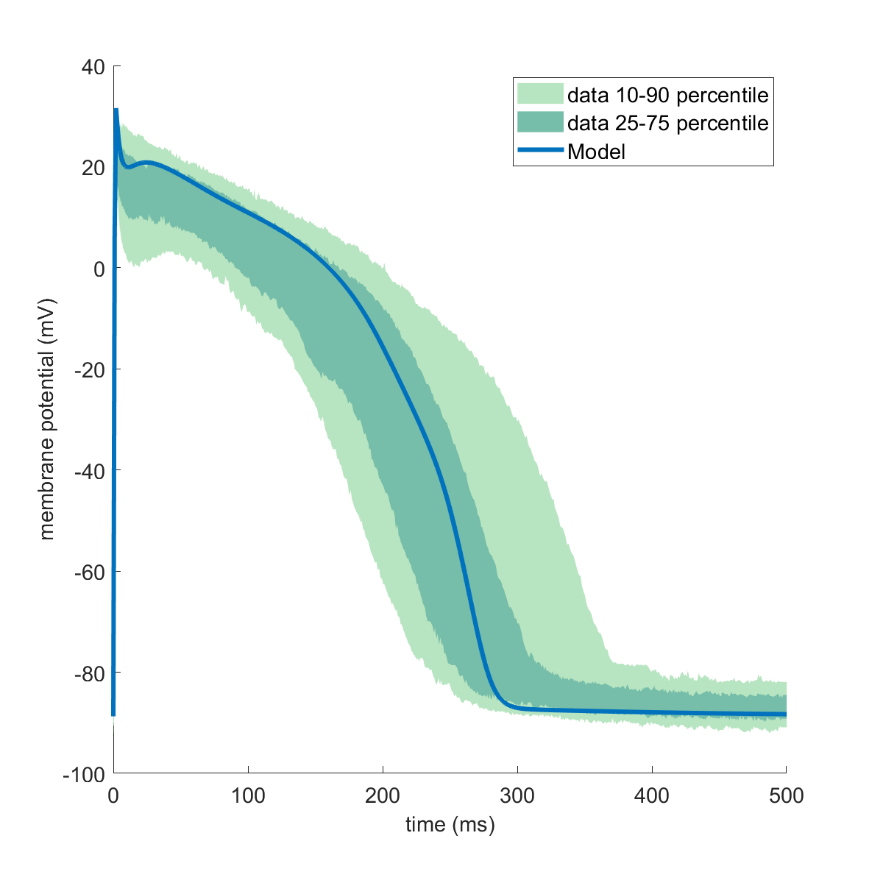


Figure S6: The new ToR-ORd-Land produced AP morphology in range with experimental data.


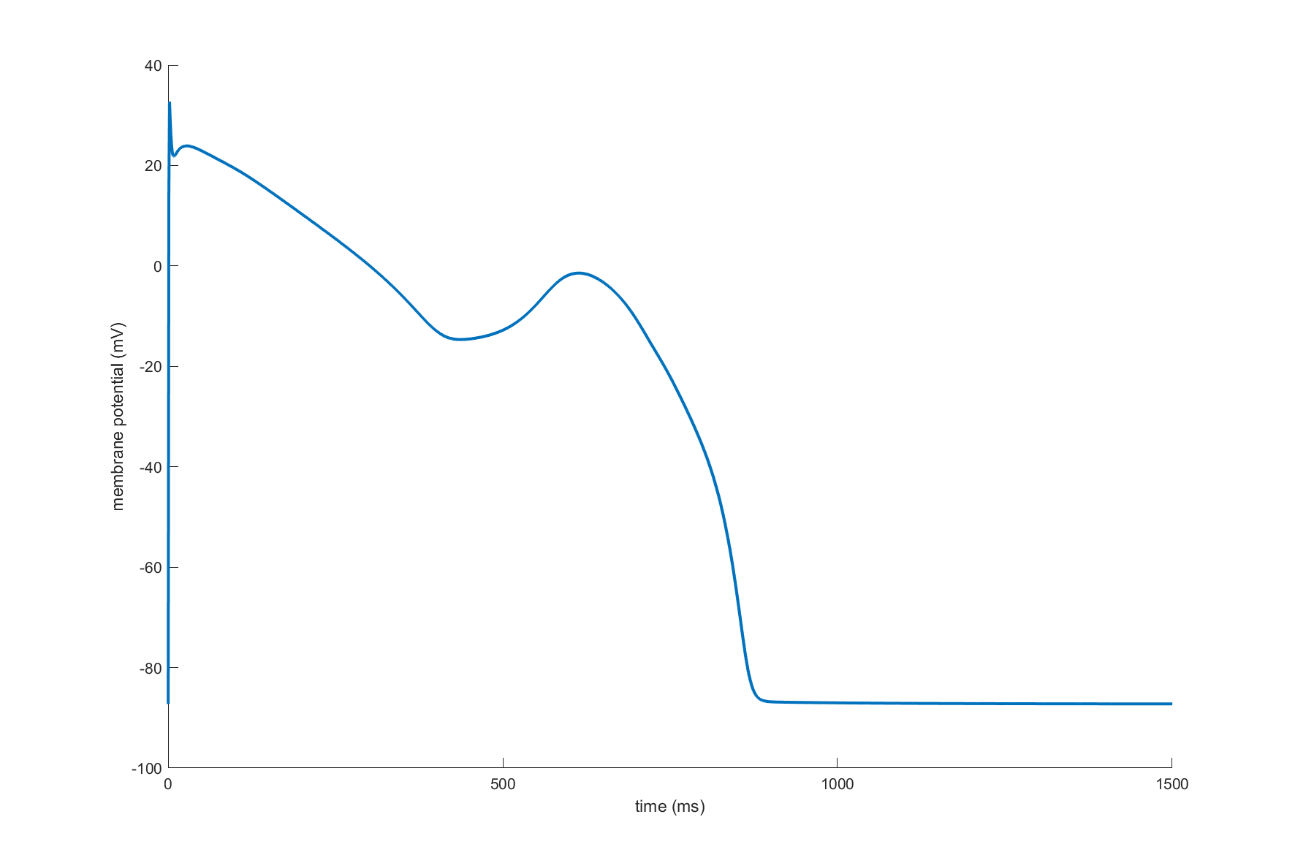


Figure S7: The new ToR-ORd-Land is able to produce EAD at 15% IKr availability at a slow pacing cycle length of 4000 ms.


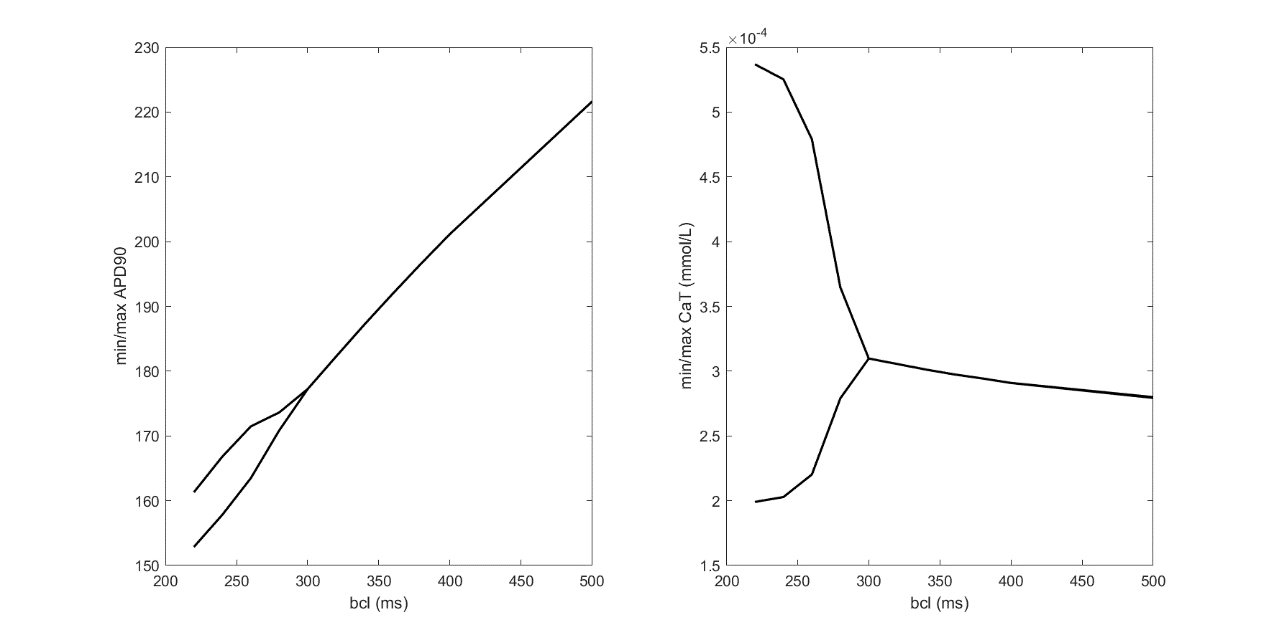


Figure S8: The new ToR-ORd-Land is able to AP and CaT alternans at fast pacing.


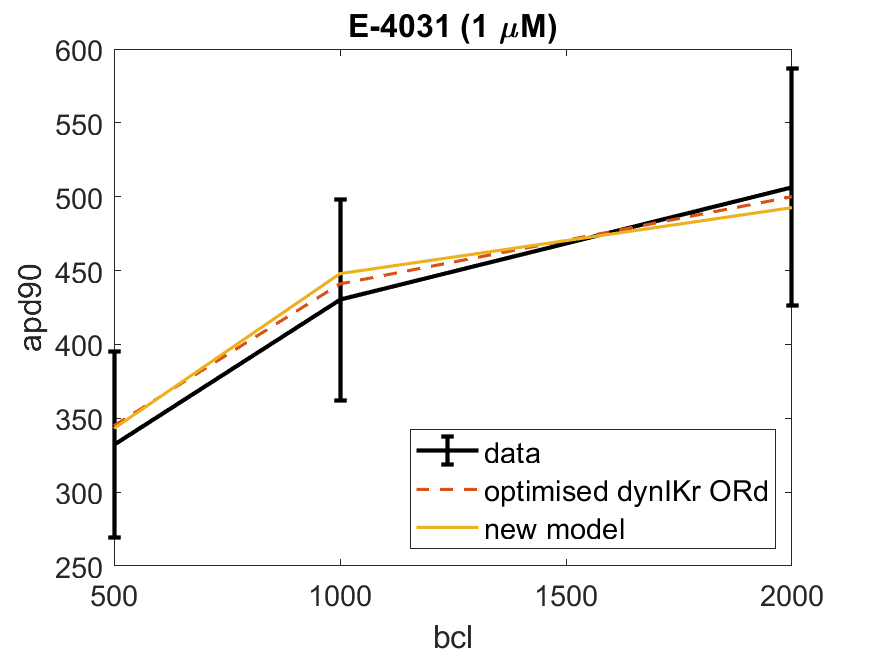

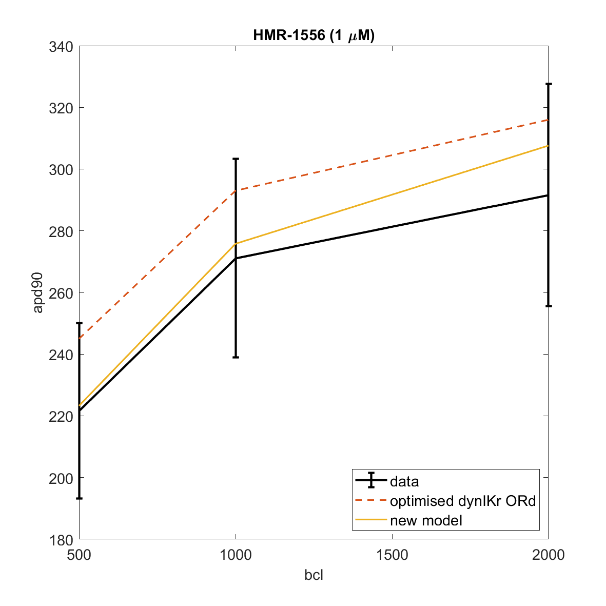

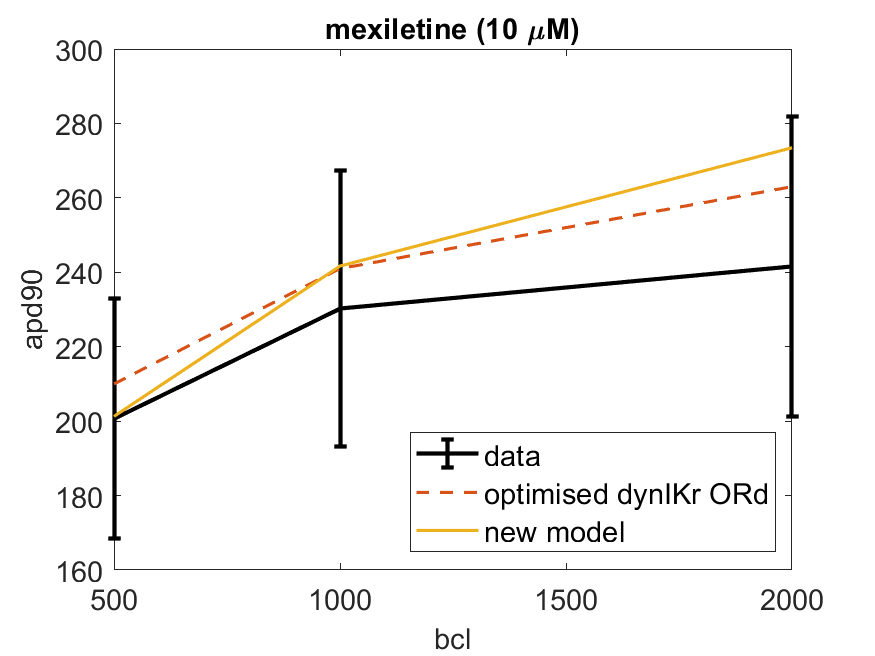

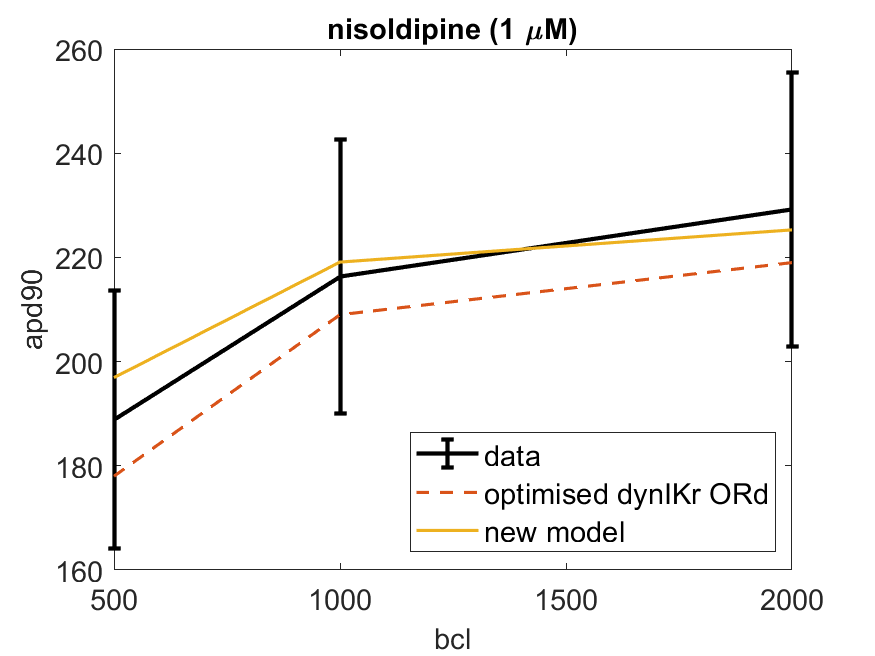


Figure S9: The new ToR-ORd-Land is able to produce action potential duration (APD) ranges consistent with experimental data under different drug actions.

**SM4: Construction, calibration and validation of the population of normal zone (NZ) and heart failure (HF) electromechanical models**

Table S1: Experimental ranges of AP, calcium transient (CaT) and active tension (Ta) biomarkers used to calibrate the NZ endocardial population of models at a pacing cycle length (CL) of 1000ms based on human cardiomyocyte experiments ^7,8,10–12^.

| **Biomarkers at 1Hz** | **minimum** | **maximum** |
| --- | --- | --- |
| **V_max_ (mV)** | 7 | 55 |
| **RMP (mV)** | -95 | -80 |
| **APD_90_ (ms)** | 180 | 440 |
| **APD_50_ (ms)** | 110 | 350 |
| **APD_40_ (ms)** | 85 | 320 |
| **APD_90_-APD_40_ (ms)** | 50 | 150 |
| **CaTD_90_ (ms)** | 220 | 750 |
| **CaTD_50_ (ms)** | 120 | 420 |
| **CaT_amp_ (mM)** | 2e-4 | 6e-4 |
| **CaT_max_ (mM)** | 2e-4 | 10e-4 |
| **CaT_min_ (mM)** | 0 | 4e-4 |
| **Ta_max_ (kPa)** | 5 | 26 |


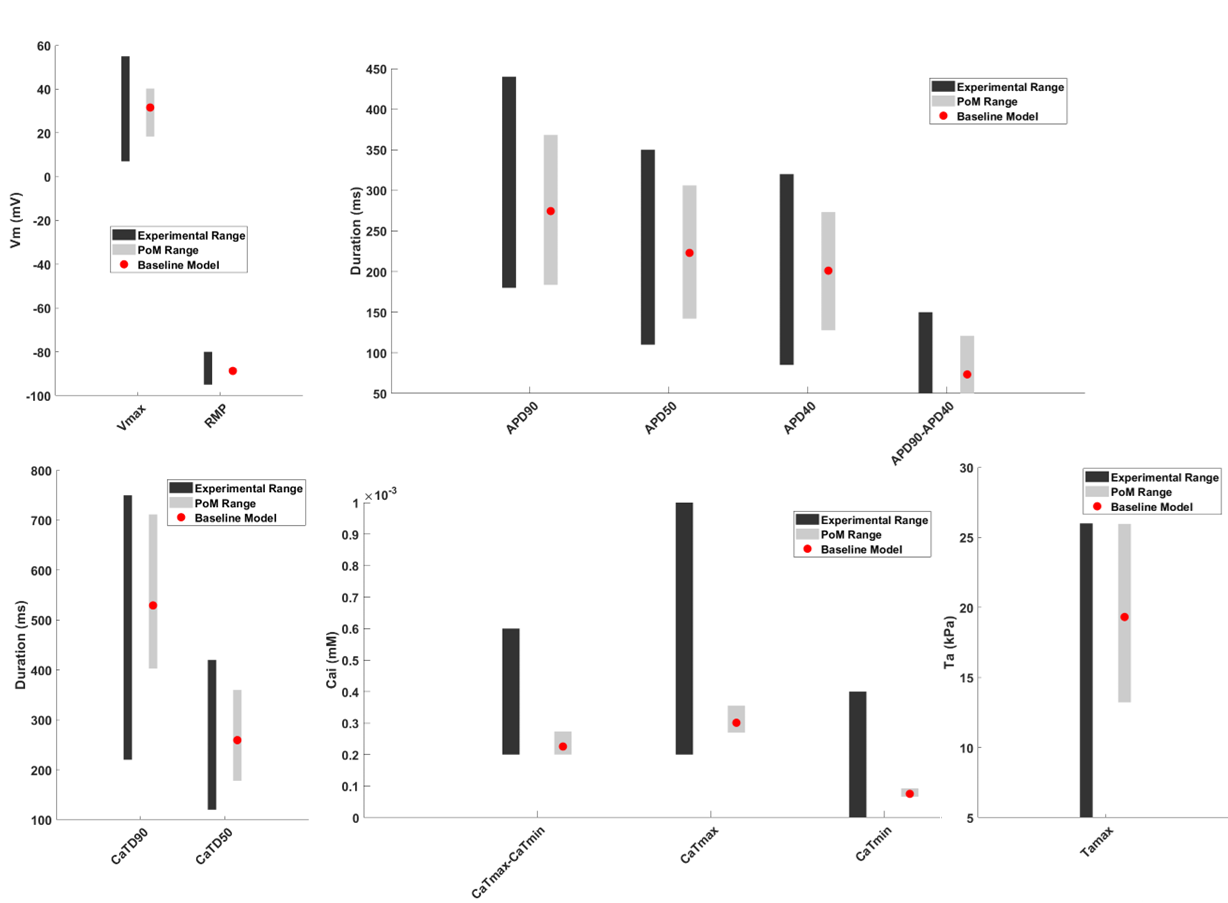


Figure S10: The accepted NZ endocardial electromechanical models produced AP, CaT biomarkers in range with experimental observations.


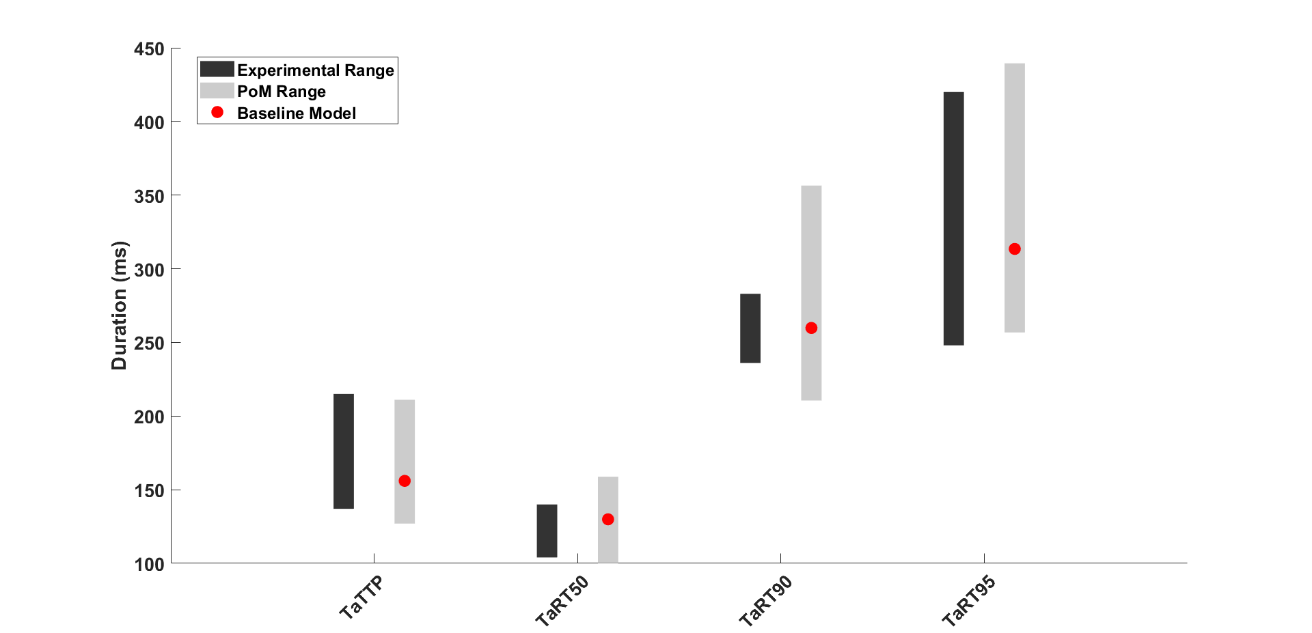


Figure S11: Although only Ta magnitude was calibrated in the endocardial NZ models, the other biomarkers of Ta kinetics were also consistent with experimental ranges.

Table S2: Experimental biomarker ratios of AP, CaT and Ta to validate the changes between NZ and HF population of models at a pacing CL of 1000ms based on human experiments of endocardial (ENDO), epicardial (EPI), and midmyocardial (MID) cardiomyocytes.

| **Biomarker at 1Hz** | **HF/NZ** | **NZ cell number** | **HF cell number** | **HF cell type** |
| --- | --- | --- | --- | --- |
| **APD_90_ EPI** ^13^ | 1.27-1.68 | 13 | 25 | Right ventricle with severe histologic and functional abnormalities^13^ |
| **APD_50_ EPI** ^13^ | 1.12-1.52 | 13 | 25 | Same as APD_90_ EPI ^13^ |
| **CaT_amp_ MID** ^14,15^ | 0.21-0.75 | 59-91 | 76-112 | Left ventricle post-myocardial-infarction at mid-stage  of HF^14^; Left ventricular end-stage HF^15^ |
| **CaT_min_ MID** ^14^ | 0.77-1.24 | 48-80 | 54-90 | Left ventricle post-myocardial-infarction at mid-stage  of HF^14^ |
| **CaTD_50_ MID** ^14^ | 0.97-1.25 | 48-80 | 54-90 | Same as CaT_min_ MID ^14^ |
| **Ta_max_ ENDO** ^7,8,12^ | 0.44-1.15 | 26 | 40 | Left ventricular end-stage  HF due to idiopathic dilated cardiomyopathy ^7,8^ or ischemic and nonischemic cardiomyopathy ^12^ |
| **TaTTP MID** ^14^ | 1.01-1.24 | 48-80 | 54-90 | Same as CaT_min_ MID ^14^ |
| **TaTTP ENDO** ^7,8,12^ | 0.89-1.27 | 26 | 40 | Same as Ta_max_ ENDO ^7,8,12^ |
| **TaRT_50_ ENDO** ^7,8,12^ | 0.9-1.35 | 26 | 40 | Same as Ta_max_ ENDO ^7,8,12^ |
| **TaRT_90_ ENDO** ^12^ | 1-1.15 | 11 | 12 | Left ventricular end-stage  HF due to ischemic or nonischemic cardiomyopathy ^12^ |
| **TaRT_95_ ENDO** ^7^ | 0.79-1.11 | 9 | 22 | Left ventricular end-stage  HF due to idiopathic dilated cardiomyopathy ^7^ |
| **Ta_min_ ENDO** ^12^ | 0.93-1.78 | 11 | 12 | Same as TaRT_90_ ENDO ^12^ |

**SM5: Contributions of individual ionic currents to HF phenotypes**


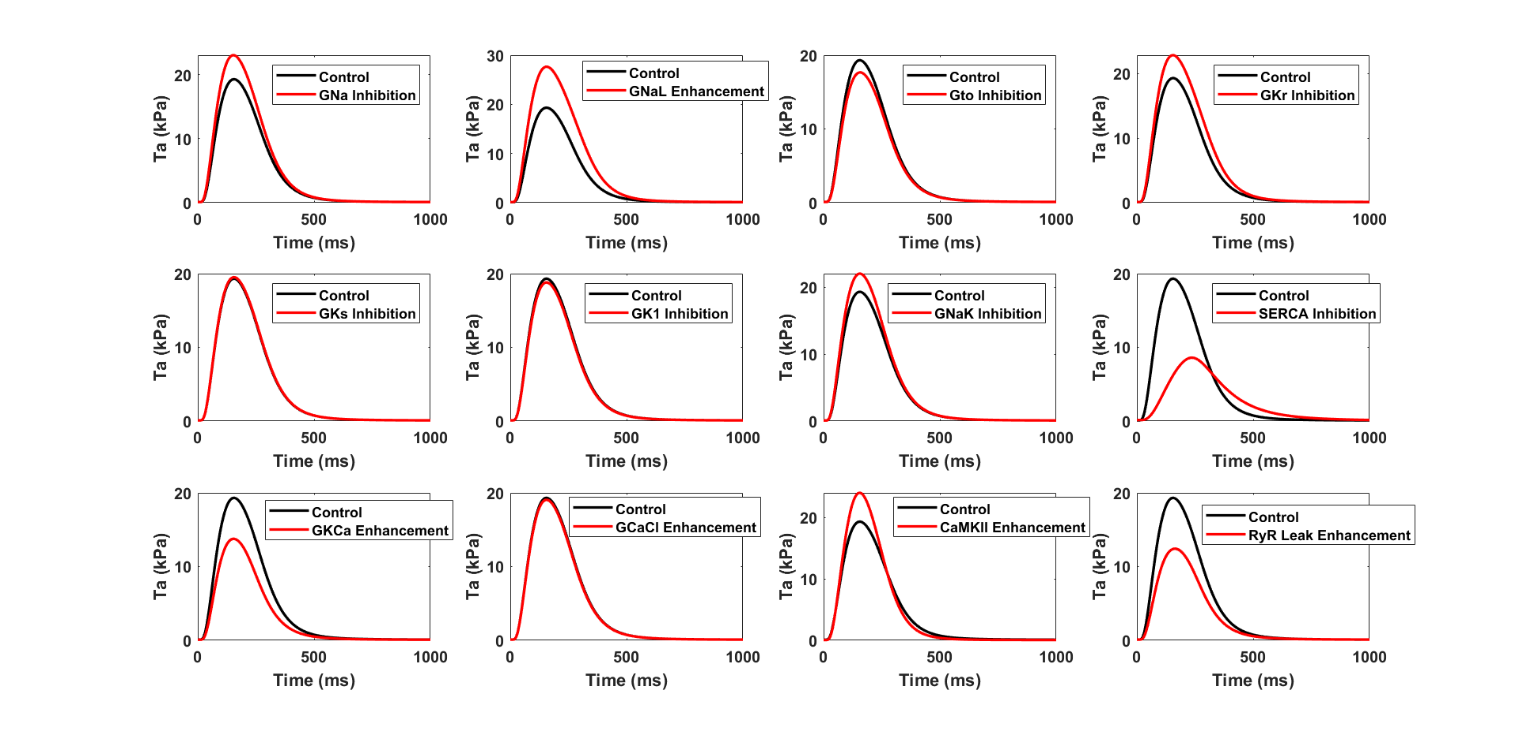

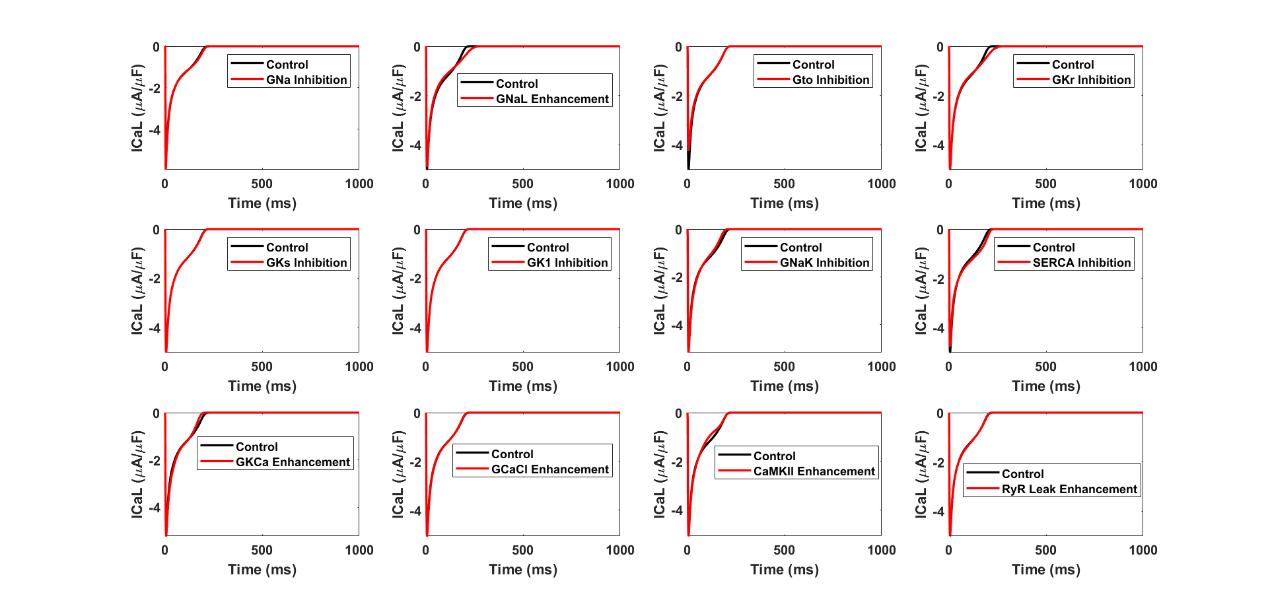


**A**

**B**

Figure S12: Effects of individual HF ionic current remodelling on the Ta (A) and the L-type calcium current (B).

Figure S13: Partial correlation coefficients (PCC) of individual ionic current conductances with AP, CaT and Ta biomarkers of endocardial HF models.


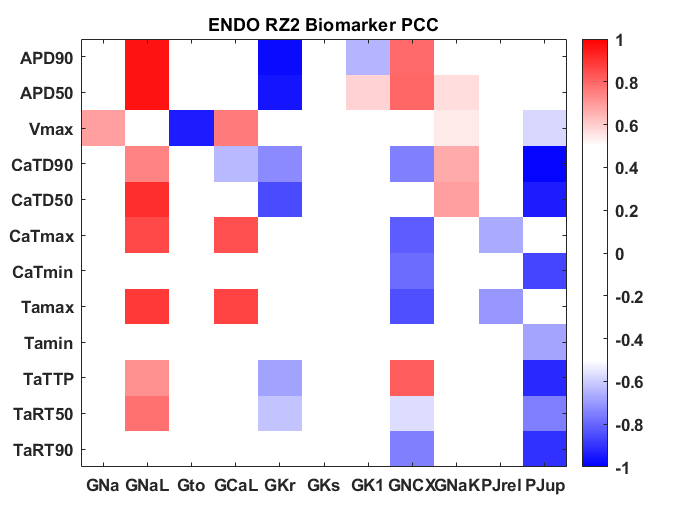

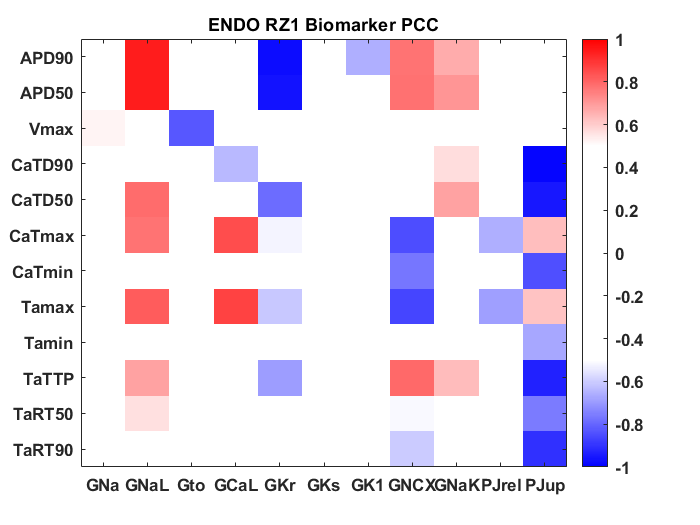

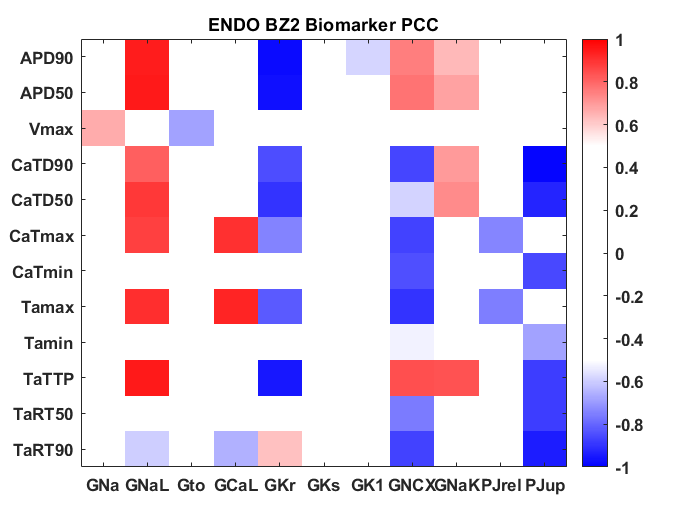

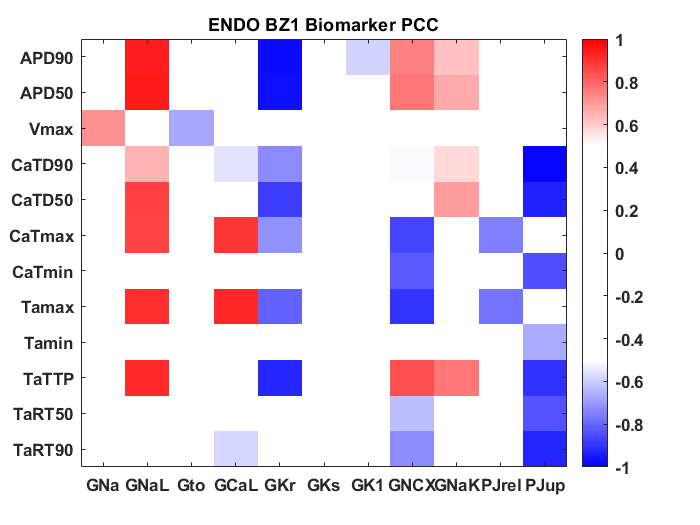


**SM6: Contributions of ionic currents to the repolarization abnormality (RA) and alternans generation in HF models**


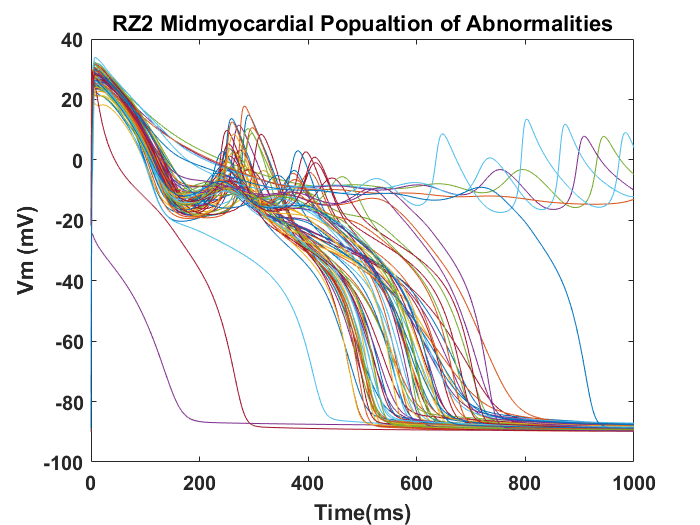

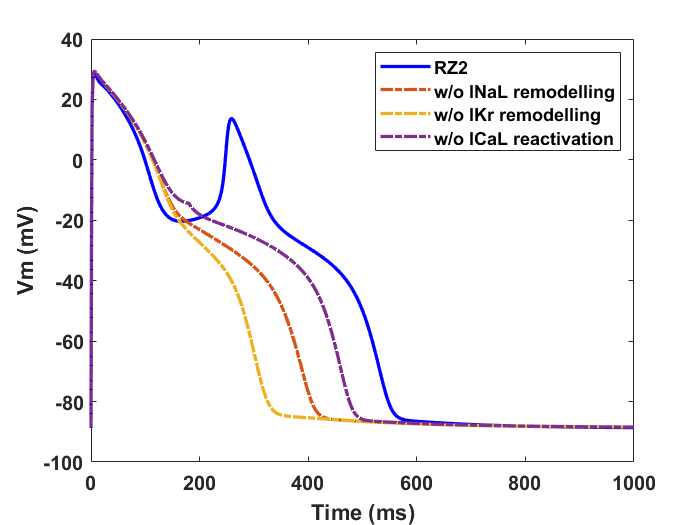


**A**

**B**

Figure S14: I_NaL_ and I_Kr_ remodelling contribute to the generation of EADs in HF models. A) Population of RZ2 midmyocardial models with EADs, repolarization failure or depolarization failure at 1Hz pacing. B) Removing either I_NaL_ enhancement or I_Kr_ suppression eliminates EAD, while terminating I_CaL_ reactivation achieves the same.


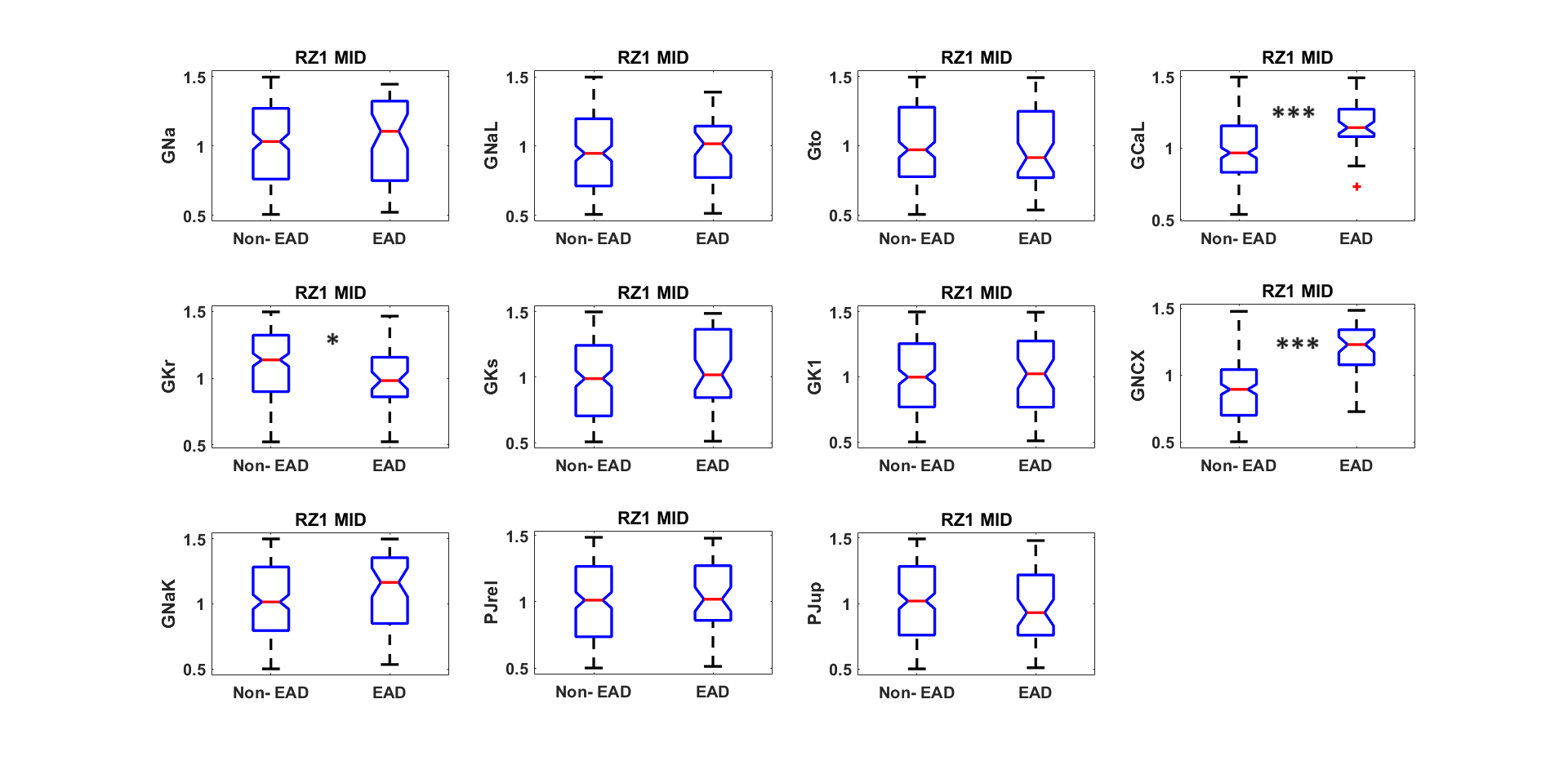


Figure S15: Parameter comparison between the Non-EAD group and EAD group in the population of RZ1 midmyocardial models. EAD models tended to have stronger G_CaL_ and G_NCX_, as well as weaker G_Kr_. (***: p<0.001, *: p<0.05)


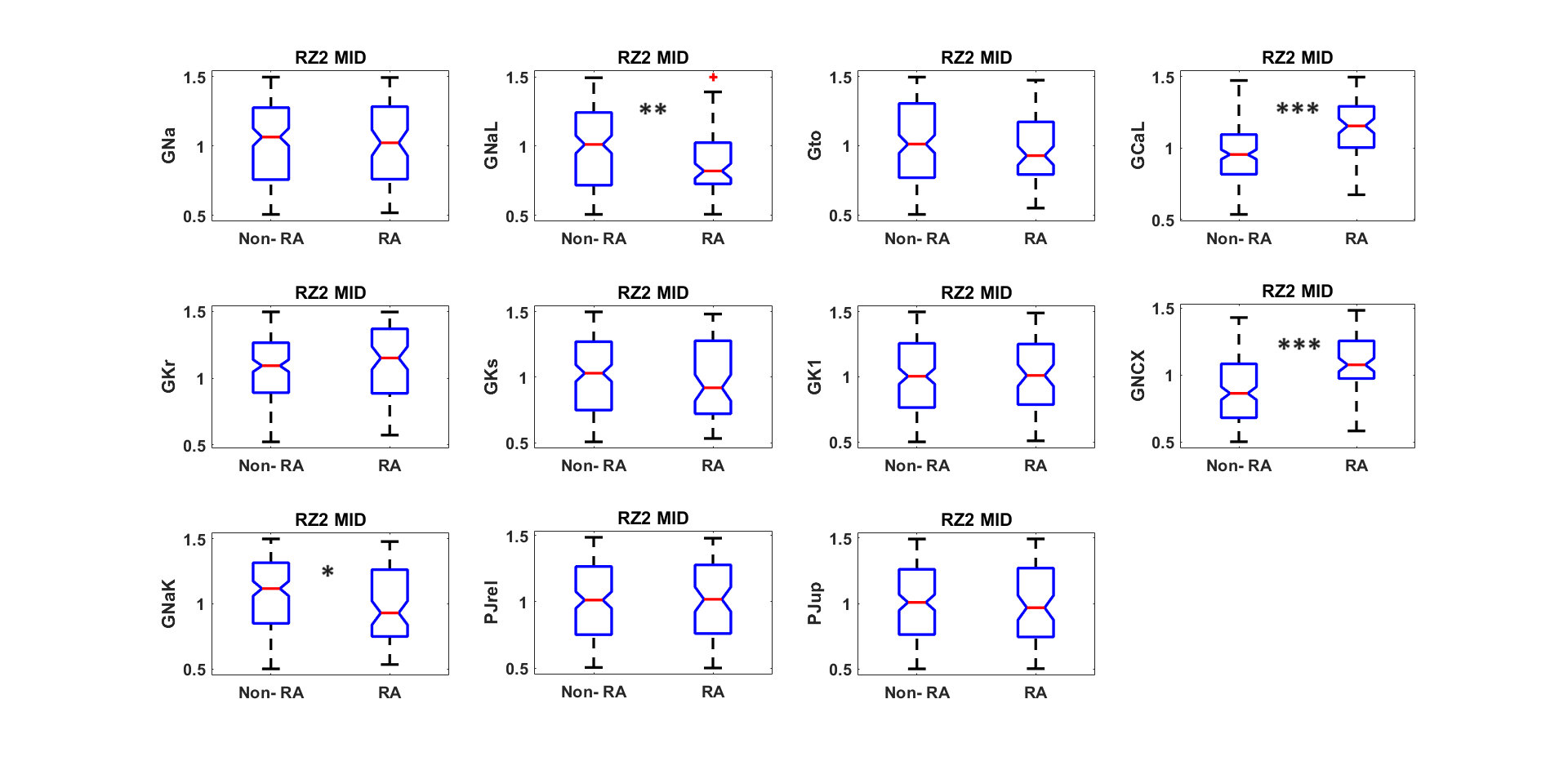


Figure S16: Parameter comparison between the Non-RA group and RA group in the population of RZ2 midmyocardial models. RA models tended to have stronger G_CaL_ and G_NCX_, as well as weaker G_NaL_ and G_NaK_. (***: p<0.001, **: P<0.01, *: p<0.05)


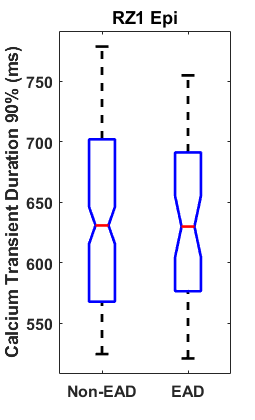

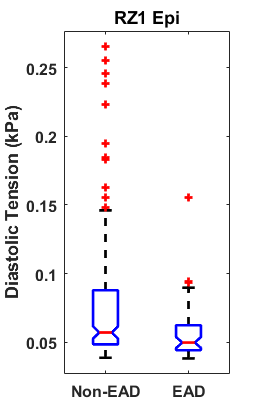

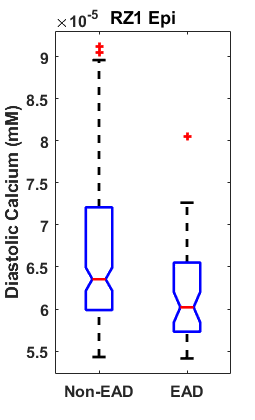

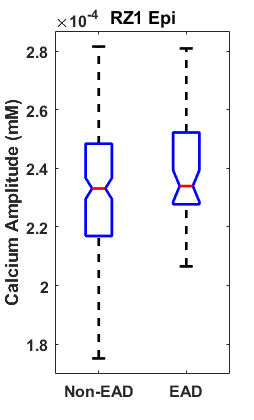

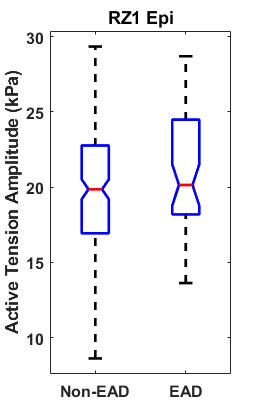

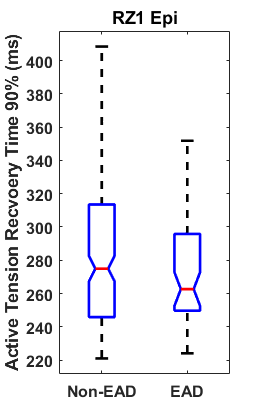


******

******

**A**

**B**

**C**

Figure S17: Comparison of the amplitudes (A), diastolic levels (B) and durations (C) of the calcium and active tension in the corresponding epicardial models with and without EAD generation in the RZ1 midmyocardial layer. (**: P<0.01)


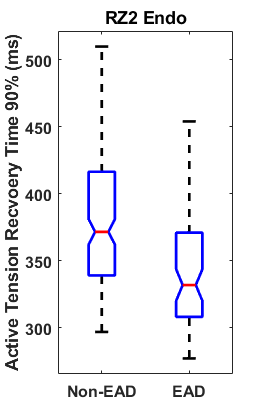

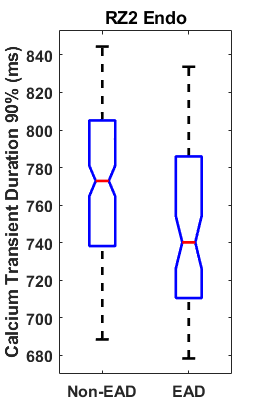

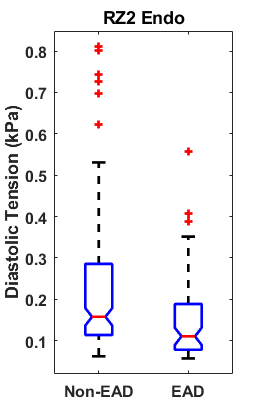

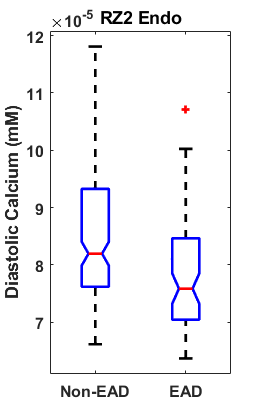

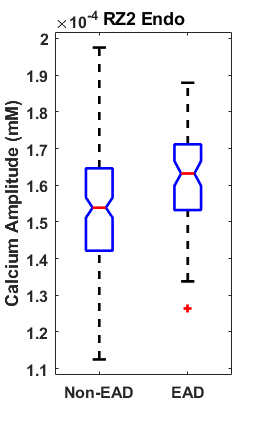

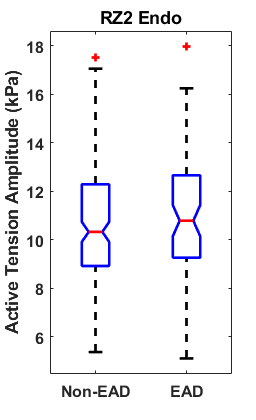


*******

*******

*******

*******

*******

**A**

**B**

**C**

Figure S18: Comparison of the amplitudes (A), diastolic levels (B) and durations (C) of the calcium and active tension in the corresponding endocardial models with and without EAD generation in the RZ2 midmyocardial layer. (***: p<0.001)


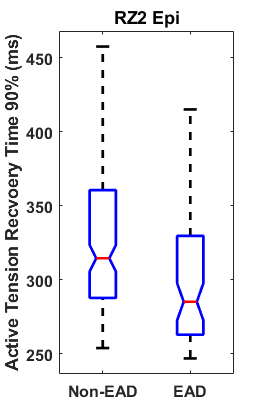

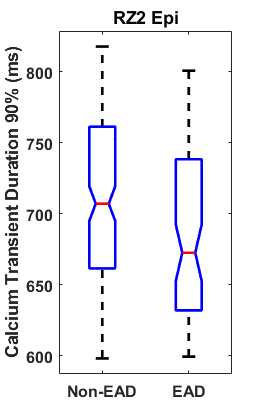

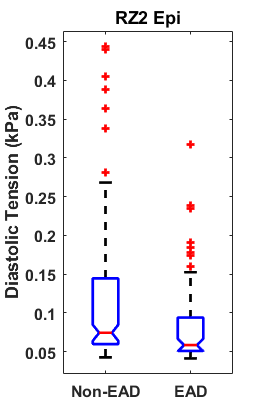

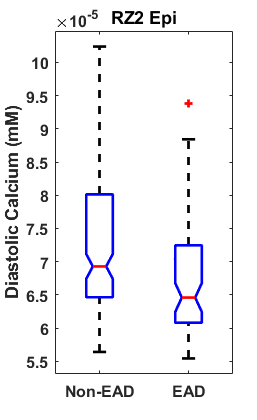

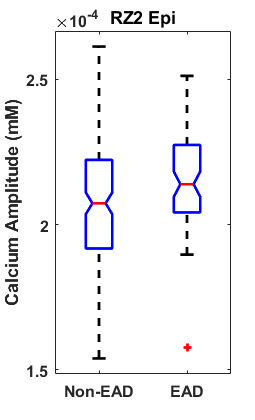

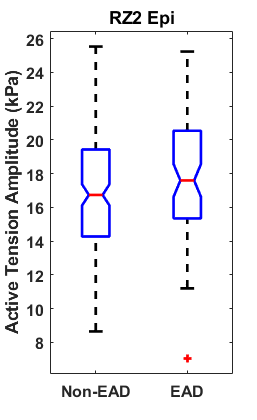


******

*******

*******

******

*******

**A**

**B**

**C**

Figure S19: Comparison of the amplitudes (A), diastolic levels (B) and durations (C) of the calcium and active tension in the corresponding epicardial models with and without EAD generation in the RZ2 midmyocardial layer. (***: p<0.001, **: P<0.01)


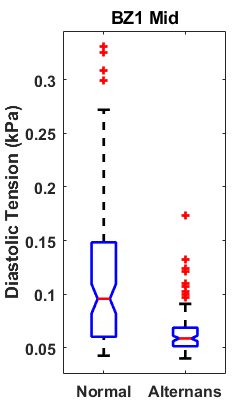

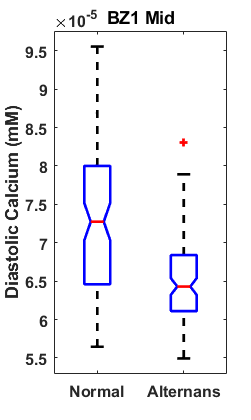

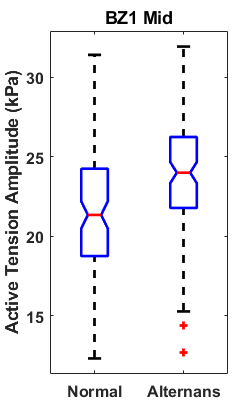

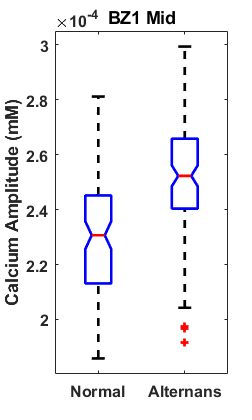

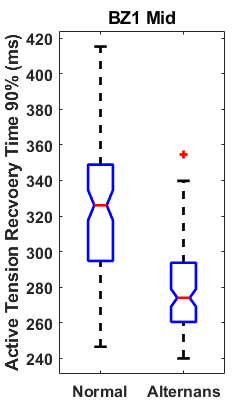

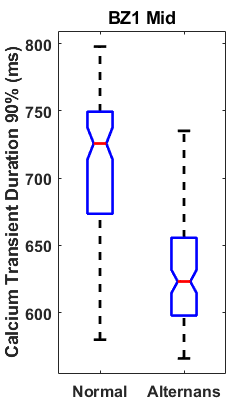

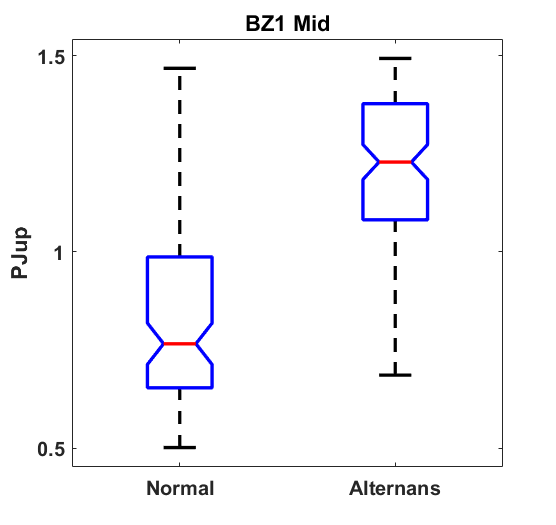


**A**

**B**

**C**

**D**

***** *** *** *****

***** *** *****

Figure S20: Comparison of the amplitudes (A), diastolic levels (B) and durations (C) of the calcium and active tension at 1Hz with and without alternans generation in the BZ1 midmyocardial HF models. D) Comparison of the P_Jup_ (SERCA) between the alternans and non-alternans models in the BZ1 midmyocardial models (***: p<0.001)

**References**

1. Chang, P. *et al.* Heterogeneous Upregulation of Apamin‐Sensitive Potassium Currents in Failing Human Ventricles. *J Am Heart Assoc* **2**, (2013).

2. Zhang, X.-D. *et al.* Coupling of SK channels, L-type Ca 2+ channels, and ryanodine receptors in cardiomyocytes. *Sci Rep* **8**, 1–13 (2018).

3. Hegyi, B. *et al.* Complex electrophysiological remodeling in postinfarction ischemic heart failure. *Proc. Natl. Acad. Sci. U.S.A.* **115**, E3036–E3044 (2018).

4. Heijman, J., Volders, P. G. A., Westra, R. L. & Rudy, Y. Local control of β-adrenergic stimulation: Effects on ventricular myocyte electrophysiology and Ca2+-transient. *J Mol Cell Cardiol* **50**, 863–871 (2011).

5. Margara, F. *et al.* In-silico human electro-mechanical ventricular modelling and simulation for drug-induced pro-arrhythmia and inotropic risk assessment. *Progress in Biophysics and Molecular Biology* **159**, 58–74 (2021).

6. Li, M. X. & Hwang, P. M. Structure and function of cardiac troponin C (TNNC1): Implications for heart failure, cardiomyopathies, and troponin modulating drugs. *Gene* **571**, 153–166 (2015).

7. Pieske, B. *et al.* Diminished post-rest potentiation of contractile force in human dilated cardiomyopathy. Functional evidence for alterations in intracellular Ca2+ handling. *J Clin Invest* **98**, 764–776 (1996).

8. Mulieri, L. A., Hasenfuss, G., Leavitt, B., Allen, P. D. & Alpert, N. R. Altered myocardial force-frequency relation in human heart failure. *Circulation* **85**, 1743–1750 (1992).

9. Rossman, E. I. *et al.* Abnormal frequency-dependent responses represent the pathophysiologic signature of contractile failure in human myocardium. *J Mol Cell Cardiol* **36**, 33–42 (2004).

10. Coppini, R. *et al.* Late sodium current inhibition reverses electromechanical dysfunction in human hypertrophic cardiomyopathy. *Circulation* **127**, 575–584 (2013).

11. Britton, O. J., Bueno-Orovio, A., Virág, L., Varró, A. & Rodriguez, B. The Electrogenic Na(+)/K(+) Pump Is a Key Determinant of Repolarization Abnormality Susceptibility in Human Ventricular Cardiomyocytes: A Population-Based Simulation Study. *Front Physiol* **8**, 278 (2017).

12. Chung, J.-H. *et al.* Impact of heart rate on cross-bridge cycling kinetics in failing and nonfailing human myocardium. *Am J Physiol Heart Circ Physiol* **317**, H640–H647 (2019).

13. Li, G.-R., Lau, C.-P., Leung, T.-K. & Nattel, S. Ionic current abnormalities associated with prolonged action potentials in cardiomyocytes from diseased human right ventricles. *Heart Rhythm* **1**, 460–468 (2004).

14. Høydal, M. A. *et al.* Human cardiomyocyte calcium handling and transverse tubules in mid-stage of post-myocardial-infarction heart failure. *ESC Heart Fail* **5**, 332–342 (2018).

15. Piacentino, V. *et al.* Cellular basis of abnormal calcium transients of failing human ventricular myocytes. *Circ. Res.* **92**, 651–658 (2003).
